# A Novel Mechanism of Programmed Cell Death in Bacteria by Toxin–Antitoxin Systems Corrupts Peptidoglycan Synthesis

**DOI:** 10.1371/journal.pbio.1001033

**Published:** 2011-03-22

**Authors:** Hannes Mutschler, Maike Gebhardt, Robert L. Shoeman, Anton Meinhart

**Affiliations:** Department of Biomolecular Mechanisms, Max Planck Institute for Medical Research, Heidelberg, Germany; Harvard University, United States of America

## Abstract

Most genomes of bacteria contain toxin–antitoxin (TA) systems. These gene systems encode a toxic protein and its cognate antitoxin. Upon antitoxin degradation, the toxin induces cell stasis or death. TA systems have been linked with numerous functions, including growth modulation, genome maintenance, and stress response. Members of the epsilon/zeta TA family are found throughout the genomes of pathogenic bacteria and were shown not only to stabilize resistance plasmids but also to promote virulence. The broad distribution of epsilon/zeta systems implies that zeta toxins utilize a ubiquitous bacteriotoxic mechanism. However, whereas all other TA families known to date poison macromolecules involved in translation or replication, the target of zeta toxins remained inscrutable. We used in vivo techniques such as microscropy and permeability assays to show that pneumococcal zeta toxin PezT impairs cell wall synthesis and triggers autolysis in *Escherichia coli*. Subsequently, we demonstrated in vitro that zeta toxins in general phosphorylate the ubiquitous peptidoglycan precursor uridine diphosphate-*N*-acetylglucosamine (UNAG) and that this activity is counteracted by binding of antitoxin. After identification of the product we verified the kinase activity in vivo by analyzing metabolite extracts of cells poisoned by PezT using high pressure liquid chromatograpy (HPLC). We further show that phosphorylated UNAG inhibitis MurA, the enzyme catalyzing the initial step in bacterial peptidoglycan biosynthesis. Additionally, we provide what is to our knowledge the first crystal structure of a zeta toxin bound to its substrate. We show that zeta toxins are novel kinases that poison bacteria through global inhibition of peptidoglycan synthesis. This provides a fundamental understanding of how epsilon/zeta TA systems stabilize mobile genetic elements. Additionally, our results imply a mechanism that connects activity of zeta toxin PezT to virulence of pneumococcal infections. Finally, we discuss how phosphorylated UNAG likely poisons additional pathways of bacterial cell wall synthesis, making it an attractive lead compound for development of new antibiotics.

## Author Summary

Most bacteria harbor toxin–antitoxin (TA) systems, in which a bacterial toxin is rendered inactive under resting conditions by its antitoxin counterpart. Under conditions of stress, however, the antitoxin is degraded, freeing the toxin to attack its host bacterium. One such TA system, PezAT, has been difficult to study in the past because the PezT toxin is so toxic without its antitoxin counterpart that bacteria die before any useful measurements can be made. Here, we use a truncated version of PezT that kills bacteria more slowly than normal, allowing us to examine the mechanisms of how this TA system operates. We find that zeta toxins convert an essential building block of bacterial cell walls (known as UNAG) into a form that prevents normal cell wall growth, causing distortions in bacterial shape that leave the bacteria vulnerable to the hydrostatic pressure of its contents. Consequently, the bacteria burst, similar to what happens when they are treated with penicillin. These results may serve useful for designing new antibiotics. Additionally, our results support the hypothesis that activation of PezT during bacterial infections may be a method by which rapidly growing bacteria can instigate a suicide program, which would promote the release of virulence factors that facilitate spread of infections.

## Introduction

Almost all prokaryotic genomes encode toxin–antitoxin (TA) systems [1]. These loci consist of bicistronic operons that encode for a bacterial toxin and its cognate inhibitor, which neutralizes the toxin under dormant conditions. Once de novo synthesis from the operon is impaired, continuous proteolytic degradation of the antitoxin eventually releases the toxin and, depending on the functional mechanism, induces cell stasis or cell death. Thus, TA systems have been linked with numerous cellular functions, including programmed cell death, maintenance of mobile genetic elements, stress response, persistence, and biofilm formation [2]–[5]. To understand how TA systems fulfill such a variety of tasks, it is crucial to unravel the molecular principles that define the mode of action of the toxins. Moreover, understanding these systems is likely to disclose new strategies for the development of antibiotic agents [6],[7].

Members of the epsilon/zeta TA family have been shown to stabilize resistance plasmids in major human pathogens such as *Streptococcus pyogenes*, *Enterococcus faecium*, and *Enterococcus faecalis* [8]–[10]. The epsilon/zeta system is encoded from a bicistronic operon, which is regulated by the repressor protein omega [11]–[13]. Upon failure of epsilon biosynthesis, the zeta toxin is released from epsilon by continuous antitoxin degradation through AAA+ proteases. Eventually, zeta becomes freed, leading to cell death [11],[14]. In addition to these plasmid-encoded epsilon/zeta systems, chromosomally encoded systems (PezAT for pneumococcal epsilon/zeta) have recently been identified on different integrative and conjugative elements of *Streptococcus pneumoniae* [15]–[17]. PezT and zeta toxins share 42% sequence identity and are structurally highly homologous [17]. In contrast, the antitoxin PezA is a multidomain protein and its C-terminal domain is similar to epsilon in its primary as well as tertiary structure. Although, the N-terminal helix-turn-helix domain of PezA acts similarly to the omega protein as a transcription repressor, the proteins are not evolutionaryily related [17]. The epsilon/zeta and the PezAT system form similar heterotetrameric complexes in which two antitoxin molecules inhibit two toxins. In addition to these pronounced structural similarities, mutational studies have shown that the two systems are functionally equivalent [17],[18].

PezAT systems are found encoded on pneumococcal pathogenicity islands that support their host with virulence factors and resistance to different antibiotics [15]–[17]. Notably, the PezT toxin of such systems was reported to accelerate the progression of pneumococcal infections, implying a role as a possible virulence factor [19]. Although epsilon/zeta systems were thought to be exclusively found in Gram-positive bacteria, recent reports have described homologous systems in Gram-negative pathogens such as *Neisseria gonorrhoeae* and enterotoxigenic *Escherichia coli* B7A [20],[21]. The broad distribution of epsilon/zeta TA systems within the bacterial kingdom suggests that they utilize a ubiquitous bacteriotoxic mechanism. Structures of zeta toxins as well as mutational studies suggested that their toxicity is connected to an ATP-dependent phosphorylation event [17],[18]. However, whereas all other TA systems known to date poison macromolecules involved in translation or replication [22]–[26], the target of the cytosolic zeta toxin family remained inscrutable [8],[14],[27].

Here, we reveal the mechanism used by zeta toxins to induce programmed cell death in bacteria. Since expression of wild-type zeta toxins leads to either instantaneous cell death or spontaneous mutation of the open reading frame [8],[17],[27], we established a system with which we could follow formation of the toxic phenotype at moderate time scales. This system enabled us to show that zeta toxins provoke an autolytic phenotype as a consequence of impaired cell wall integrity and breakdown of the osmotic barrier. We found that zeta toxins represent a novel family of kinases that manipulate a central metabolic branch point of bacterial cell wall synthesis. The crystal structure of a zeta toxin bound to its target allowed us to map the enzyme–substrate interactions and revealed that the kinase activity is indeed responsible for the toxic function in vivo. In fact, we were able to show that the phosphorylated product inhibits MurA, the enzyme responsible for the first step of peptidoglycan synthesis in bacteria.

## Results

### PezT Expression Kills Cells during Division

**Figure 1:**
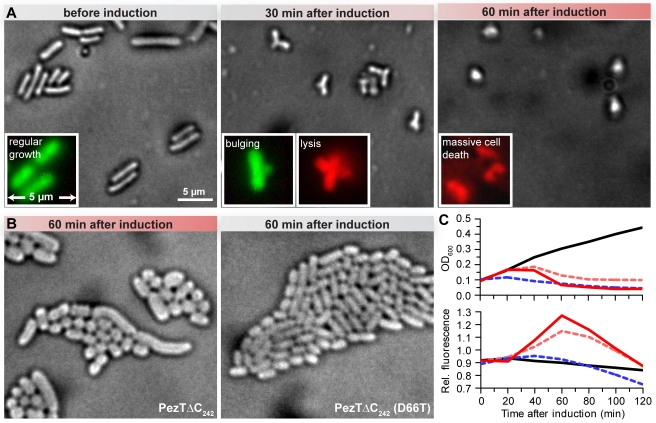
Phenotype of PezTΔC_242_ expression in *E. coli*. (A) Phase contrast image of fixed *E. coli* cells bearing pET28b(*pezTΔC_242_*) grown in liquid cultures before and 30 min and 60 min after toxin expression. The insets show representative unfixed cells that were examined after live/dead fluorescence staining. (B) Phase contrast image of adherently growing *E. coli* cell 1 h after induction of PezTΔC_242_ (left) or PezTΔC_242_ (D66T) expression (right). (C) Time correlation between cell growth/lysis and the breakdown of the osmotic barrier. Growth kinetics were monitored by measuring the optical density (upper panel) of cultures expressing PezTΔC_242_ (solid red line) or nontoxic PezTΔC_242_ (D66T) (solid black line). The influx of membrane-impermeable propidium iodide present in the medium was measured in parallel by the increase in fluorescence at 620 nm (lower panel). Additional cultures expressing nontoxic PezTΔC_242_ (D66T) were treated with either 50 µg/ml ampicillin (dashed red line) or 15 µg/ml tetracycline (dashed blue line).

Genetic manipulations of the full-length zeta or homologous PezT toxins without the cognate antitoxin are unfeasible because of the high toxicity of the proteins. Moreover, toxin variants that can be isolated from surviving clones are generally inactive because of spontaneous mutations [8],[17]. We found that a carboxy-terminally truncated variant lacking the last 11 amino acids (PezTΔC_242_, henceforth referred to simply as PezT) can be stably maintained in *E. coli*. Importantly, sequencing of the plasmid DNA isolated from *E. coli* cells after prolonged expression experiments showed that this variant did not accumulate any spontaneous mutations. Nevertheless, this variant still retained the toxic phenotype. Half an hour after induction of PezT expression, we found that the cells in such cultures formed midcell-positioned bulges following membrane permeabilization and lysis (Figures 1A, S1A, and S1B). Notably, cells that had survived to that point were apparently unable to undergo cytokinesis, even though chromosome replication was complete (Figure S1B). One hour after induction, massive cell death had occurred and the few intact cells that remained were characterized by small size and an ovoid morphology (Figure S1C). To exclude the possibility that the observed phenotype is due to general overexpression, we performed control experiments with cells bearing the expression plasmid of the nontoxic variant PezT (D66T) [17]. We did not observe bulge formation or lysis after induction of PezT (D66T) (Figure S1D). Therefore, we concluded that the observed phenotype was caused by the specific action of PezT.

### PezT Inhibits Cell Wall Synthesis

Lysis through bulge formation implied that cells poisoned by PezT suffered from defects in their cell wall integrity. Bacterial growth and binary fission demand a tightly controlled balance between murein synthesis and degradation. Perturbations of this balance are known to cause uncontrolled peptidoglycan degradation by murein hydrolases following lysis because of the intracellular turgor pressure [28]. Since PezT appeared to kill cells predominantly prior to cytokinesis, which requires the build-up of septal murein, we speculated that the toxin targets cell wall synthesis. To test this hypothesis, we induced toxin expression in adherently growing cultures, which are less exposed to osmotic and mechanic stress than liquid cultures [29]. One hour after induction, we observed that these cells adopted a bloated, misshapen morphology that was clearly different from the normal rod-like shape of cells expressing nontoxic PezT (D66T) (Figure 1B). In fact, these cells resembled spheroplasts that form after treatment with β-lactam antibiotics such as ampicillin, which are authentic inhibitors of bacterial peptidoglycan synthesis [30].

To further corroborate our hypothesis that PezT targets cell wall synthesis, we probed whether toxin expression resembles β-lactam treatment in general. We found that the onset of cell death caused by PezT expression was preceded by a strong rise in membrane permeability, by measuring influx kinetics of the membrane-impermeable dye propidium iodide (Figure 1C). Eventually, membrane permeability increased enough to allow the 31-kDa periplasmatic ribonculease I to enter the cytosol, resulting in massive rRNA degradation (Figure S1E). Strikingly, we could provoke almost identical characteristics of cell death in *E. coli* expressing nontoxic PezT (D66T) by additional treatment of the culture with ampicillin (Figures 1C and S1F). In contrast, we did not observe any of these symptoms when cells were treated with tetracycline, which solely targets protein biosynthesis (Figures 1C and S1F).

In summary, we concluded that the toxic mechanism of PezT causes inhibition of cell wall synthesis that eventually provokes bacterial autolysis. Nevertheless, the identity of the molecular target of PezT remained enigmatic, since inhibition of cytosolic steps of bacterial cell wall synthesis can occur on a multitude of levels [31]. However, recent reports had shown that the zeta protein from *S. pyogenes* is toxic in eukaryotes such as *Saccharomyces cervisiae* [32]. This suggested the presence of a putative ubiquitous substrate whose modification by the toxins corrupts a shared metabolic branch point found in prokaryotes and eukaryotes. Such a fundamental metabolite is uridine diphosphate-*N*-acetylglucosamine (UNAG), which is produced in the hexosamine biosynthesis pathway and is found in all kingdoms of life [33]. This nucleotide sugar is essential for the formation of a plethora of glycoconjugates, among them the peptidoglycan macromolecule in prokaryotes. We next set out to determine whether UNAG is indeed the substrate of PezT.

### Zeta Toxins Phosphorylate the Cell Wall Precursor UNAG

**Figure 2:**
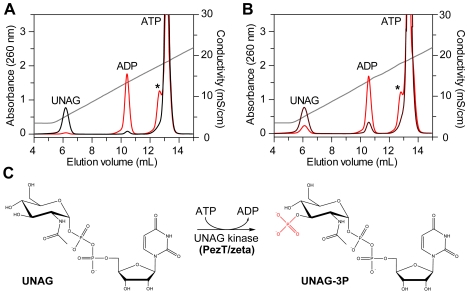
PezT from *S. pneumoniae* and zeta from *S. pyogenes* phosphorylate UNAG. (A) Samples containing 0.25 mM UNAG, 5 mM MgCl_2_, 1 mM ATP, and 1 µM PezTΔC_242_ (red) or additionally 1 µM PezA antitoxin (black) were analyzed by anion exchange chromatography after 1 h of incubation at 25°C. The asterisk indicates the retention volume of the product formed by PezTΔC_242_ in the absence of the antitoxin PezA. (B) Analysis of the equivalent reaction using 1 µM epsilon/zeta complex from *S. pyogenes* after 1 h (dark red) and 24 h (red) of incubation. The asterisk indicates the retention volume of the product formed by zeta. Note that UNAG turnover was slow because of the presence of stoichiometric amounts of the antitoxin epsilon. However, in contrast to the PezAT system, zeta is not entirely inhibited. This difference is most probably caused by the different TA affinities in the two TA systems [11],[46]. (C) Schematic reaction mechanism of UNAG-3P formation by zeta toxins.

Given the structural similarity of PezT and zeta toxins with phosphotransferases [17],[18], we speculated that they modify UNAG by phosphorylation. Therefore, we purified our toxic PezT variant from poisoned *E. coli* and probed its activity in vitro. Indeed, using anion exchange chromatography, we observed an ATP- and Mg^2+^-dependent modification of UNAG (Figures 2A and S2A–S2C). In addition to adenosine diphosphate formation, we noticed a product that was more negatively charged than the substrate UNAG and that eluted close to the remaining ATP. This strongly suggested that the PezT toxin had phosphorylated UNAG. We could exclude that the observed UNAG modification was the result of a contaminating activity, since addition of stoichiometric amounts of the cognate antitoxin PezA completely inhibited turnover of both UNAG and ATP (Figures 2A and S2B). Further, we showed that the PezT activity was specific for the presence of the 2′-*N*-acetyl group on the sugar moiety and the stereoisomeric form of UNAG, since selectivity for uridine diphosphate (UDP)–glucose and UDP-*N*-acetylgalactosamine was dramatically reduced (Figure S2E and S2F). Most importantly, we also showed that the zeta toxin from *S. pyogenes* can catalyze the same reaction as PezT and modifies UNAG using ATP (Figure 2B). This strongly suggests that the enzymatic function described is a conserved activity of the entire family of zeta toxins.

We investigated next whether PezT indeed phosphorylates UNAG. To this end, we performed electrospray ionization spectrometry experiments, which showed that the product of the PezT toxin and UNAG differ by the mass of a phosphoryl group (Δ*m/z* = 80; Table S1). Using fragmentation by tandem mass spectrometry we could cleave the phosphorylated UNAG molecule into two main fragments, one with a mass corresponding to UDP and one corresponding to *N*-acetylglucosamine with a phosphoryl group attached to a hydroxyl group (Table S1). Ultimately, we showed by nuclear magnetic resonance (NMR) (Figure S3) that the PezT toxin had attached a phosphoryl group to the 3′-hydroxyl group of the *N*-acetylglucosamine moiety. In conclusion, we have demonstrated that zeta and PezT toxins are UNAG kinases that form UDP-*N*-acetylglucosamine-3′-phosphate (UNAG-3P) from UNAG using ATP and Mg^2+^ as a cofactor (Figure 2C).

### Toxicity Is Caused by the UNAG Kinase Activity

Site directed mutagenesis studies of PezT and zeta toxins yielded several inactive toxin variants that have been linked with binding of the at-that-time-unknown substrate [17],[18]. We therefore were interested in a structural characterization of a toxin bound to the substrate. Previous structural reports on epsilon/zeta and PezAT complexes showed that the toxin is structurally similar to poly- and mononucleotide kinases [17],[18]. Based on these findings, the active site of zeta and PezT toxin has been identified and a mechanism of toxin inhibition has been proposed. Once bound to the antitoxin, side chain groups of the antitoxin block the ATP binding site [17],[18]. The second substrate binding site, however, remains unaffected by the antitoxin. Thus, we surmised that PezT and zeta toxins are still able to bind UNAG even if they are inhibited by the antitoxin. Indeed, we could determine the crystal structure of the epsilon/zeta TA complex from *S. pyogenes* bound to UNAG at 2.7 Å resolution (Figure S4; Table S2).

**Figure 3:**
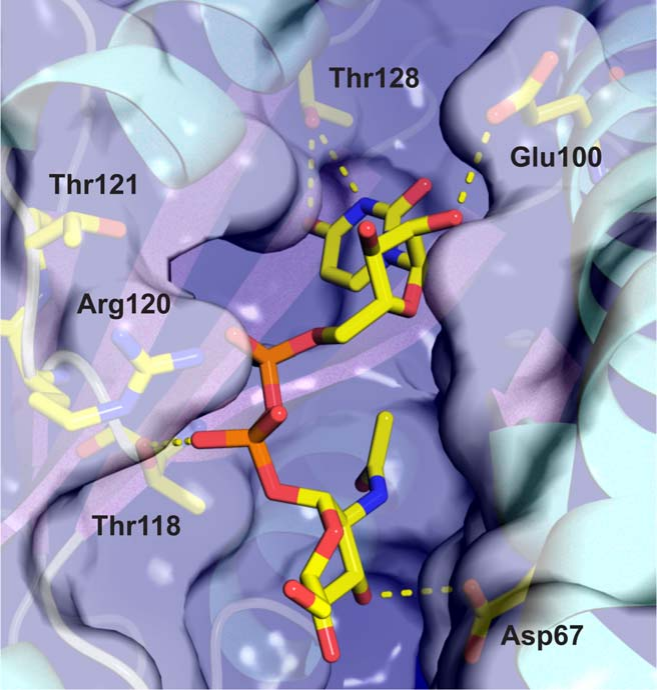
UNAG bound to zeta toxin from *S. pyogenes*. A transparent molecular surface representation of zeta toxin with a ribbon representation shown underneath. Residues of the zeta toxin that are important for substrate binding are depicted as a stick model. UNAG shown as a stick model is embedded in a deep cleft. Hydrogen bonds relevant for substrate binding are illustrated as yellow dashed lines.

UNAG binds to a deep cleft at the molecular surface of the zeta toxin (Figure 3). The side chain group of Asp67 forms a hydrogen bond (3.2 Å) to the 3′-hydroxyl group of the amino sugar moiety of UNAG. Based on superposition with structurally related phosphotransferases, this particular side chain of the zeta toxin had been suggested to be the catalytic base, which deprotonates the substrate [18]. For PezT toxin, Asp66 is the functionally equivalent residue, and, as mentioned above, its mutation to a threonine residue totally abolishes toxicity. Since we could not detect any activity of PezT (D66T) in our kinase assay (Figure S2D), we conclude that the phosphoryltransfer reaction is required for the bacteriotoxic mechanism of zeta toxins. Thr118 of the zeta toxin formed a hydrogen bond with an oxygen atom of the β-phosphate group of UNAG (2.8 Å), and exchange of this conserved residue in the PezT toxin to a valine residue also yielded a nontoxic variant [17]. The conserved Thr121, which is in close proximity to the bound UNAG but did not contact the substrate directly, has been shown to interfere with toxicity of PezT only mildly [17]. Additional specific contacts between the zeta toxin and UNAG are electrostatic interactions of Arg120 with the phosphate groups of UDP. Apart from van der Waals interactions, specific hydrogen bonds are formed between Thr128 and the uracil base and between Glu100 and the 2′-hydroxyl group of the UDP ribose. Most likely, mutations of these residues will affect toxicity as well. Thus, our data show that UNAG binding by the zeta toxin is mediated by residues that yield nontoxic and kinase-deficient zeta and PezT variants in vivo and in vitro.

### UNAG-3P Enriches during PezT Poisoning In Vivo and Inhibits Peptidoglycan Synthesis

**Figure 4:**
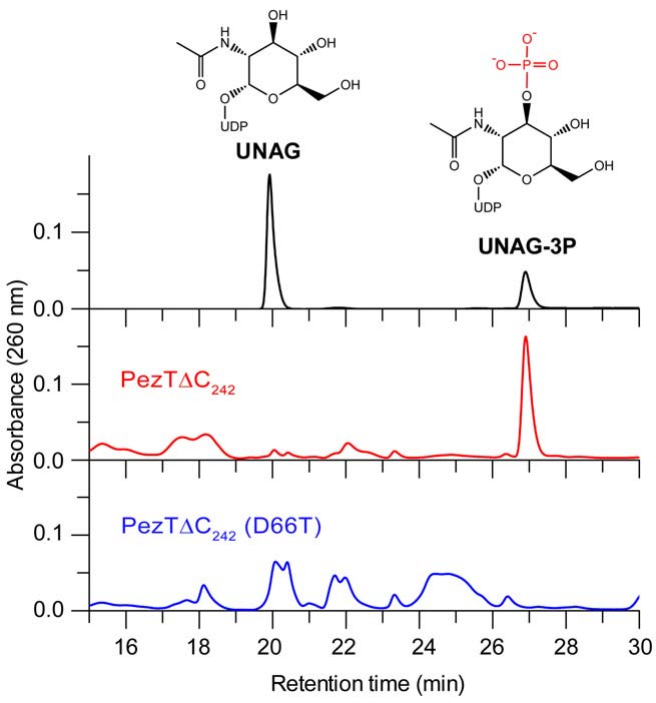
UNAG-3P accumulates in cells expressing PezTΔC_242_. In vivo extracts of metabolites from cells after 1 h of PezTΔC_242_ (red) or PezTΔC_242_ (D66T) (blue) expression. The mixture was analyzed by HPLC using a strong anion exchange column. The chromatogram of authentic standards is shown in the top panel (black). Note that individual concentrations of isolated small molecules cannot be compared quantitatively, since concentrations of individual runs were adjusted to similar absorbance at 260 nm. Furthermore, some species may have been partially degraded during extraction.

In accordance with our in vitro results, we also showed that UNAG-3P is the main product of the PezT toxin activity in vivo. In fact, we identified enriched UNAG-3P in low-molecular-weight-metabolite pools of PezT-poisoned cells obtained by aqueous acetonitrile extraction and HPLC (Figure 4). We confirmed the presence of UNAG-3P in these fractions by chromatography of authentic standards (Figure 4) and by mass spectrometry (Table S1). Our experiments suggested that cells that contained active PezT toxin constantly accumulated UNAG-3P, whereas the pool of UNAG was largely depleted.

**Figure 5:**
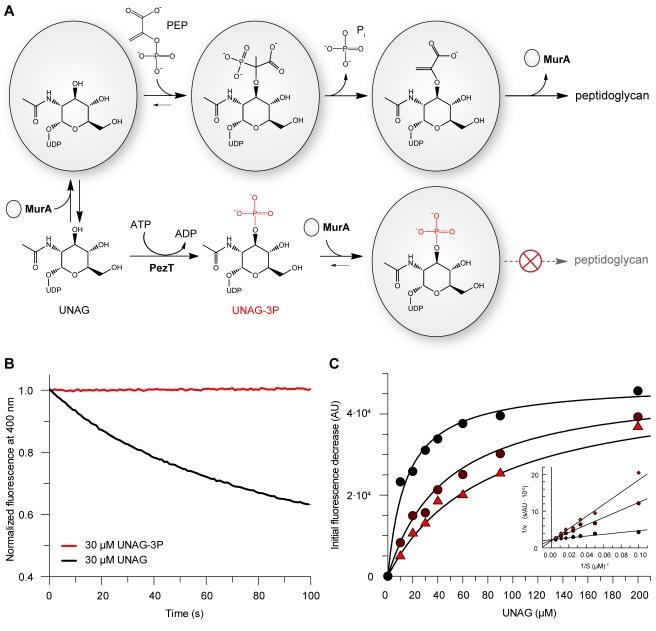
Mechanism of MurA inhibition by UNAG-3P. (A) MurA performs the first step of UDP-muramic acid biosynthesis. After sequential binding of UNAG and phosphoenolpyruvate (PEP), a tetrahedral intermediate is formed that yields enolpyruvyl-UNAG after cleavage of inorganic phosphate. UNAG-3P most likely mimics this tetrahedral intermediate and thereby inhibits MurA catalysis by competitive inhibition. (B) MurA is able to transfer the enolpyruvyl moiety from phosphoenolpyruvate (1 mM) to UNAG (black) but not UNAG-3P (red). MurA enzyme kinetics were followed by coupling the reaction to phosphate-dependent cleavage of fluorescent 7-methylguanosine by nucleoside phosphorylase, resulting in a decrease in fluorescence at 400 nm (λ_exc_ = 300 nm). (C) Determination of the *K*_i_ of UNAG-3P for MurA under steady state conditions. The UNAG-3P concentrations for each saturation curve were 0 µM (black circles), 15 µM (dark red circles), and 30 µM (red triangles). A Lineweaver-Burk plot is shown as inset. The saturation curves were fitted globally by nonlinear regression assuming competitive inhibition (black line), yielding a *K*_m_ of 15 µM for UNAG and a *K*_i_ of 7 µM for UNAG-3P.

Modification of the amino sugar 3′-hydroxyl group by PezT and zeta toxins suggests several possible scenarios by which UNAG-3P can interfere with peptidoglycan synthesis. One of these is inhibition of the conserved enolpyruvyl transferase MurA, which catalyzes the initial step of muramic acid synthesis. Subsequent to the hexosamine biosynthesis pathway, MurA modifies the amino sugar 3′-hydroxyl group of UNAG (Figure 5A) and thereby provides the starting point for peptidoglycan biosynthesis [34].

MurA catalyzes the transfer of the enolpyruvyl moiety of phosphoenolpyruvate to the 3′-hydroxyl group of *N*-acetylglucosamine, forming enolpyruvyl-UNAG and inorganic phosphate (Figure 5A). Due to modification of the 3′-hydroxyl group by the phosphate group, we speculated that MurA would be unable to utilize UNAG-3P, which renders the phosphorylated nucleotide sugar unusable for peptidoglycan synthesis. Indeed, we did not observe any MurA-dependent turnover of UNAG once it had been phosphorylated by PezT in an in vitro activity assay designed to measure the release of inorganic phosphate (Figure 5B).

During the native reaction of MurA, a negatively charged tetrahedral adduct is formed [35]. Because of the resemblance of the MurA tetrahedral intermediate and UNAG-3P (Figure 5A), we hypothesized that UNAG-3P could bind to MurA in a fashion similar to that of the normal substrate UNAG. In this hypothesis zeta toxins would not only produce a stable dead-end metabolite but would also halt cell wall synthesis directly by inhibition of MurA. In fact, we found that increasing UNAG-3P concentrations decreased the apparent turnover rate of UNAG by MurA (Figure S5). This strongly suggests that UNAG-3P binds to MurA, forming an inactive, non-productive complex. Furthermore, we observed that increasing UNAG-3P concentrations led to an increase of the apparent *K*_m_ of MurA for UNAG without affecting the maximal reaction rate *V*_max_ when we analyzed the initial rate of product formation at varying UNAG and UNAG-3P concentrations (Figure 5C). This implies that the phosphate group of UNAG-3P mimics the tetrahedral intermediate during MurA catalysis and is a reversible inhibitor that eliminates turnover of phosphenolpyruvate via the modification of the 3′-hydroxyl group of the *N*-acetylglucosamine moiety. The Michaelis-Menten dataset could be fitted globally by a competitive inhibition mechanism that yielded a *K*_i_ of 7 µM for UNAG-3P in the presence of physiological concentrations of phosphoenolpyruvate. (Figure 5A). Thus, zeta toxins not only produce a metabolic dead-end product but additionally form a competitive inhibitor for peptidoglycan synthesis.

### Slow Growth Protects Cells from Toxin-Induced Autolysis

**Figure 6:**
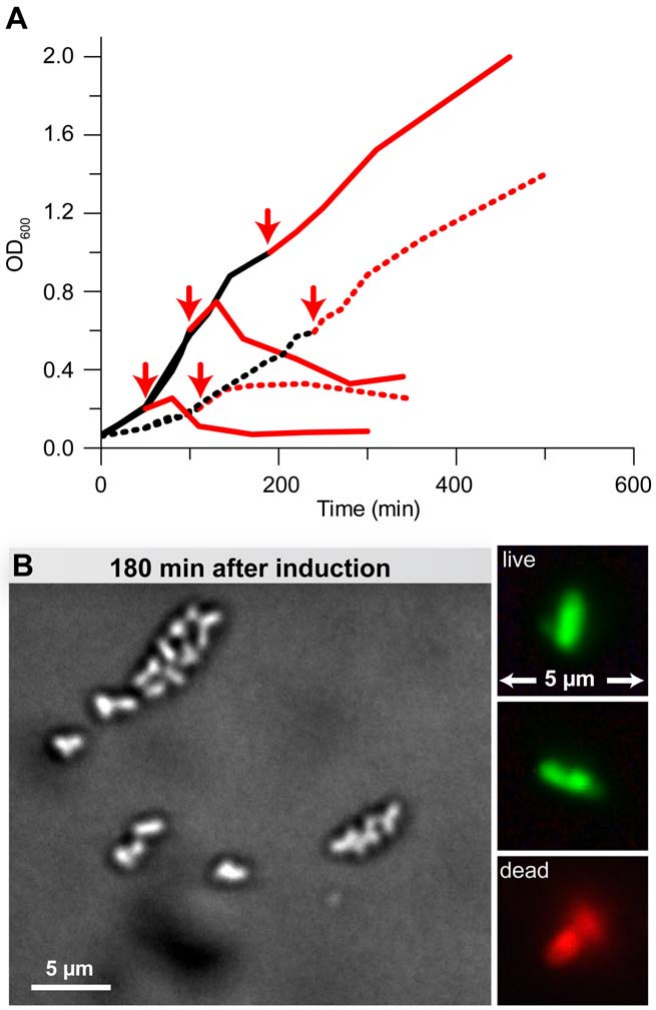
Growth rate determines toxicity of PezT expression. (A) Cell growth monitored by optical density measurements of parallel cultures after PezTΔC_242_ induction (red arrows) at different optical density. Growth in fresh LB medium (solid) and nutritionally deprived LB (dashed) is shown before (black) and after induction (red) with IPTG at the optical densities indicated. (B) Cells grown in exhausted medium after 180 min of PezTΔC_242_ expression observed by phase contrast microscopy after fixation or after fluorescent live/dead staining (inset). Note that the culture was induced at an optical density at which cell growth continues upon PezT expression.

Most antibiotics that target peptidoglycan synthesis are known to rapidly kill bacteria during exponential growth but fail to kill slowly growing and stationary cells [36]. Therefore, we considered whether slow growth was also an option to evade cell killing through the mechanism utilized by zeta toxins. When we induced toxin expression at low optical densities, where the general growth rate was rapid, we observed the lytic phenotype described above. In contrast, induction of PezT in cells approaching stationary phase did not result in lysis (Figure 6A). This indicated that UNAG-3P was predominantly toxic for fast-dividing cells, whereas the toxic effect was less severe for cells proliferating more slowly. In order to provoke slow growth, we grew cells in a nutritionally deprived medium. Generally, cells growing in such a medium were much smaller and had an increased doubling time (Figures 6A and S6A). As expected, lysis caused by PezT expression in nutritionally deprived medium was less severe and observable only after induction at the lowest optical density tested (OD_600_ = 0.2) (Figure 6A). We could exclude that this finding was caused by differential expression levels of PezT as the cytosolic concentrations of the recombinant protein were comparable to those of cultures grown in fresh Luria broth (LB) medium (Figure S6B). When we inspected cells of cultures surviving toxin expression in nutritionally deprived medium 3 h after induction, the majority of cells had accumulated membrane protrusions and lost their characteristic rod-like shape (Figure 6B). Thus, PezT was still performing its disruptive activity within peptidoglycan metabolism. However, under the growth conditions tested, the damage done to the cell wall was not sufficient for autolysis of the majority of cells. Therefore, we conclude that minimizing the demand on cell wall precursors through small size and slow growth enables bacteria to survive toxin action for an extended period of time.

## Discussion

The mechanism of how epsilon/zeta TA systems kill their host cell has remained a mystery to date [8],[14],[27]. In this study, we demonstrate that expression of PezT induces defects in cell wall integrity and eventually causes bacterial autolysis in *E. coli* cells. We show that PezT and zeta toxins define a previously unknown family of kinases that phosphorylate the nucleotide sugar UNAG with high specificity at the 3′-hydroxyl group of the *N*-acetylglucosamine moiety. We found that this modification blocks further utilization of the nucleotide sugar for muropeptide synthesis and thereby creates a stable dead-end product that accumulates intracellularly. We further showed that the formation of UNAG-3P yields a potent inhibitor of the enzyme MurA, which synthesizes the first committed step of bacterial cell wall synthesis. Thus, zeta toxins and PezT are to our knowledge the first toxins of TA systems that can be shown to directly target cell wall synthesis. This explains our finding that PezT causes death of primarily fast-growing *E. coli* cells requiring significant murein synthesis during elongation and division.

### Additional Points of Attack for PezT and Zeta Toxins

The synthesis of muramic acids is regulated by a negative feedback loop in which MurA is inhibited by its downstream product UDP-*N*-acetylmuramic acid in *E. coli* [37]. Thus, PezT and zeta toxin activity impairs muramic acid synthesis in two different ways: first, it depletes the pool of UNAG precursors and, second, UNAG-3P is a competitive inhibitor of MurA. Thus, increasing UNAG-3P levels favor UNAG-3P production by inhibition of the competing murein synthesis pathway. UNAG-3P will interfere with peptidoglycan synthesis in Gram-negative and Gram-positive organisms, since MurA is highly conserved among prokaryotes [34]. Furthermore, since UNAG is an essential precursor for teichoic and lipoteichoic acid synthesis in Gram-positive bacteria [38], formation of UNAG-3P is also likely to be detrimental to their synthesis. UNAG phosphorylation will also compete with lipid A synthesis of Gram-negative bacteria, since condensation of the lipid anchor and UNAG is performed via the amino sugar 3′-hydroxyl group of UNAG. Thus, zeta toxins ubiquitously interfere with synthesis of a variety of cell wall components, independent of the cell wall architecture, and cells harboring an epsilon/zeta or PezAT TA system are provided with a potent killing system.

### Are PezT and Zeta Toxins Always Fatal to the Host?

UNAG is an abundant metabolite in bacteria [39], and we therefore do not expect the activity of PezT and zeta toxins to immediately kill the host. This is in agreement with the previous finding that depending on the dose and exposure time to free toxin, its activity either leads to growth arrest or cell lysis [14]. We argue that the toxin acts as a killer when the toxin activation rages out of control. Such a situation is realized when, for instance, a plasmid encoding an epsilon/zeta system TA is lost and the zeta toxin is released via degradation of the epsilon antitoxin. Moreover, we speculate that the bactericidal action of zeta toxin release will be higher during fast growth, when cells are more vulnerable to inhibition of cell wall synthesis. This matches our finding that cells react differently to the toxin activity, depending on the environmental conditions. We surmise that cells can readjust their cell wall metabolism during situations where toxin activation is temporary and reversible, for instance during transcriptional or translational arrest. Our in vivo experiments suggest that a subpopulation of cells can adapt to the drain in cell wall precursor components by adopting a state of dwarfism paired with slow growth. Such different scenarios can explain the unresolved ambiguity as to why zeta toxins perform postsegregational killing in one situation or trigger cell stasis in another [8],[14].

### How Can PezT Be Beneficial to the Host?

**Figure 7:**
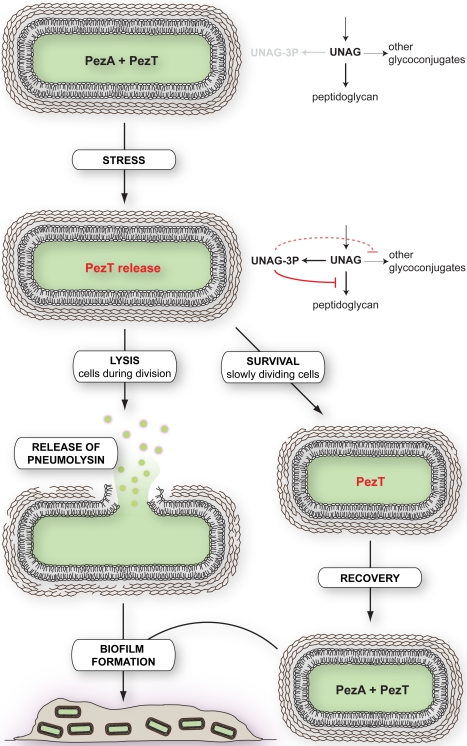
Model for the function of the pneumococcal PezAT systems during infection. Stress conditions lead to release of the UNAG kinase PezT via degradation of the antitoxin PezA. PezT converts the cellular pool of UNAG to UNAG-3P, which leads to inhibition of peptidoglycan synthesis and competes with the synthesis of other glycoconjugates. Metabolically silent persister cells as well as slowly dividing cells will survive PezT release. In contrast, cells that require fully functional murein synthesis, such as rapidly dividing cells, will undergo lysis and release cytosolic pneumolysin, a major virulence factor of *S. pneumoniae* [55]. Moreover, partial autolysis and the general inhibition of capsular polysaccharide synthesis by UNAG-3P will favor biofilm formation. Cells surviving PezT release will eventually recover by production of PezA in absence of the stress conditions.

The pneumococcal PezT toxin has been suggested to support virulence of its pathogenic host during infection, since strains in which the PezT gene has been deleted are attenuated in mouse models of infection [19]. One of the major pneumococcal virulence factors that accelerate infection progress is the pore-forming toxin pneumolysin [40],[41]. Intriguingly, pneumolysin is located in the cytosol of *S. pneumoniae* and requires a bacteriolytic activity in order to be released into the lumen [42],[43]. It is therefore tempting to speculate that pneumococcal strains bearing a PezAT gene cassette are equipped with an additional option to trigger lysis and pneumolysin release in a subpopulation of cells (Figure 7).

Release of PezT toxin from PezA antitoxin might appear under conditions that lead to either prolonged down-regulation of protein biosynthesis or enhanced PezA degradation. Similar to other TA systems, down-regulation of PezA biosynthesis could occur, for instance, during antibiotic treatment or amino acid starvation, when general transcription or translation is impaired [44]. Under these conditions, constant proteolytic degradation of PezA by constitutive proteases would eventually activate PezT. On the other hand, different TA systems within a single host were shown to be specifically activated under different stress conditions [45]. Whereas in case of the epsilon/zeta system, constant degradation of epsilon by the constitutive protease Lon and ClpXP [14] leads to toxin activation, PezT release seems not to be performed by a housekeeping protease [46]. Thus, it is rather likely, that activation of the PezAT system in its host organism is tightly controlled and requires a specific event such as stress response [46].

The ability of TA systems to induce cell lysis or cell stasis has also been linked to biofilm and persister cell formation in pathogens [47]. Biofilm formation of *S. pneumoniae*, for example, is thought to be initiated by the formation of cell aggregates that require autolysis of a subpopulation [48],[49]. Most probably, moderate activation of PezT and zeta toxins will support autolysis of rapidly dividing cells, and thus PezT and zeta toxins can be beneficial for the entire cellular population despite the suicide of individual cells. Cells with reduced metabolism and growth, such as persisters, might survive the toxins' activity and are thus selected (Figure 7). Additionally, UNAG-3P formation is also likely to interfere with hyaluronic acid capsulation of streptococci, which needs to be down-regulated and reduced during biofilm formation [50],[51], since condensation of the polysaccharide in this pathway is linked via the amino sugar 3′-hydroxyl group of UNAG. Eventually, survivors will be protected in a biofilm and can recover from stress conditions and toxin activation by synthesis of new cognate antitoxin.

### Concluding Remarks

UNAG-3P is a suicide antibiotic, because bacteria are harmed by their self-inflicted enzymatic activity depending on environmental conditions. This is in contrast to common antibiotics that bacteria produce and target against other species. Additionally, UNAG-3P is a naturally derived lead compound for the development of novel antibiotics. Our results imply that either activation or inhibition of epsilon/zeta and PezAT systems will interfere with the fate of the host bacteria and thus make them a potent Achilles' heel for microbes.

## Materials and Methods

### In Vivo Characterization of Poisoned *E. coli* Cells

For microscopy, cultures of *E. coli* BL21-CodonPlus (DE3)-RIL (Stratagene) bearing pET28b(*pezTΔC_242_*) or pET28b(*pezTΔC_242_(D66T)*) were grown in 100 ml of LB medium supplemented with kanamycin (50 µg/ml) and chloramphenicol (34 µg/ml) at 37°C in unbaffled flasks with mild shaking. Protein expression was induced by addition of IPTG to 1 mM at an OD_600_ of 0.4. To evaluate the effects of toxin expression on cell membrane integrity, the BacLight live/dead bacterial viability kit (Molecular Probes) was used according to the supplier's instructions. Phase contrast and fluorescence images were captured with a ProgRes C3 CCD camera (Jenoptic) on an Axiovert 135 microscope (Zeiss) using an oil immersion objective lens (100×/N.A. 1.3). The viability of the culture was assessed using filters with 450-nm to 490-nm excitation and 520-nm long pass emission for green fluorescence (live) or 545-nm band pass excitation and 590-nm long pass emission for red fluorescence (dead). To additionally improve visualization of membrane defects, 200 µl of culture was withdrawn and immediately fixed in 1 ml of a 1∶3 (*v/v*) mixture of acetic acid and methanol. Cells were pelleted by centrifugation, resuspended in 0.9% (*w/v*) NaCl, and spotted on an LB agar slab. Subsequently, cells were inspected by phase contrast microscopy using an Axiovert 405M (Zeiss) equipped with a 100×/N.A. 1.25 oil immersion objective lens. Adherent cultures were derived from a liquid culture grown overnight and surviving PezTΔC_242_ expression. Cells were spotted on a microscope slide covered with LB agar and incubated for 1 h at 37°C. Protein expression was induced by diffusion of IPTG into the agar drop sandwiched between cover slides and subsequently incubated for 1 h at 37°C before inspection. Unfixed cells were inspected directly by phase contrast microscopy as described above. Equivalent growth experiments using preconditioned LB medium are described in Text S1.

To measure time-resolved breakdown of the osmotic barrier, cells were grown in LB medium at 37°C to an OD_600_ = 0.2 in absence of any inducing agent. Next, 75 µl of culture was diluted with an equal volume of LB containing 1 mM IPTG and 20 µM propidium iodide. Control cultures expressing PezT (D66T) were additionally supplemented with ampicillin (50 µg/ml) or tetracycline (15 µg/ml). Samples for baseline correction were not inoculated with bacteria. All samples were transferred to a black 96-well microtiter plate (Corning) and were incubated at 37°C on a Thermomixer comfort (Eppendorf) equipped with a MTP sample holder. Breakdown of the osmotic barrier and staining of cytosolic DNA/RNA was monitored by measuring fluorescence at 620 nm in a Varioskan Flash (Thermo Scientific) with fluorescence excitation set to 520 nm at 20-min intervals. The optical density was recorded from the same samples in absorbance mode. Between measurements, the microtiter plates were covered with an AirPore tape sheet (Qiagen). For each time series, fluorescence intensities were baseline corrected and averaged (*n* = 6). Experiments monitoring breakdown of the osmotic barrier by breakdown of ribosomal RNA are described in Text S1.

### PezT/Zeta Nucleotide Sugar Kinase Assay

The kinase activity of PezTΔC_242_ and its inhibition by PezA were investigated by incubating 1 µM recombinant toxin in buffer R (25 mM HEPES-NaOH [pH 7.5], 100 mM NaCl, 5 mM MgCl_2_) supplemented with 1 mM ATP and 0.25 mM of the nucleotide sugars UNAG, UDP-*N*-acetylgalactosamine, or UDP-glucose at 25°C for the times mentioned in the respective figure captions (Figures 2 and S2). All nucleotide sugar species were purchased from Sigma. After incubation, samples were diluted 1∶2 with H_2_O, filtered and applied to a 1-ml MonoQ column (GE Healthcare) equilibrated in 50 mM Tris-HCl (pH 8.0) at 8°C. The different nucleotide/nucleotide sugar species were eluted with a gradient to 1 M NaCl. Inactivity of the nontoxic variant PezTΔC_242_ (D66T) was confirmed by performing the same assay using 3 µM protein. Since inhibition of the *S. pyogenes* zeta toxin by the epsilon antitoxin is much weaker than in the PezAT system found in *S. pneumoniae* [11],[46], we could verify the phosphoryltransfer reaction of the zeta toxin directly by extended incubation (24 h) of 1 µM purified complex with the reaction mixture described above. Quantitative production of UNAG-3P as well as electrospray ionization tandem mass spectrometry and NMR experiments verifying its identity are described in Text S1.

### Crystallization and Structure Determination of the Epsilon/Zeta/UNAG Complex

The epsilon/zeta complex was purified as described previously [18]. The complex was crystallized using the hanging-drop vapor diffusion method by mixing 1 µl of protein—12 mg/ml protein in buffer (25 mM NaCl, 50 mM Tris-HCl [pH 7.5])—and 1 µl of reservoir solution containing 0.2 M sodium acetate (pH 4.8), 16% (*w/v*) PEG 1500, and 7% (*v/v*) 2-methyl-2,4-pentanediol. Crystals were soaked for 30 min in mother liquor supplemented with 10 mM UNAG and flash cooled in liquid nitrogen. Diffraction data were collected at the Swiss Light Source (Villigen, Switzerland), beamline X10SA at 100 K. Phases were refined by molecular replacement methods with REFMAC [52] using the apo-structure of epsilon/zeta (Protein Data Bank accession number 1GVN) as starting model. Detailed descriptions of the methods applied are found in Text S1. Atomic coordinates and structure factor amplitudes have been deposited in the Protein Data Bank (http://www.pdb.org/) under accession number 3Q8X.

### In Vivo Detection of UNAG-3P

*E. coli* cells were grown as described for the microscopy experiments. Expression of the PezT proteins was induced at an OD_600_ of 0.4 for 1 h. Cells were harvested by centrifugation and resuspended in ice-cold 80% (*v/v*) aqueous acetonitrile solution. The suspension was incubated for 30 min on ice and with regular periods of gentle agitation. Cells were pelleted by centrifugation (20 min, 11,000*g*, 4°C), and the supernatant shock frozen in liquid nitrogen and stored at −80°C. For HPLC analysis, the extract was concentrated by vacuum concentration and resuspended in deionized water. Insoluble compounds were removed by high-speed centrifugation and filtration with Ultrafree-MC centrifugal filter units (Millipore). The A_260_ of the extracts was adjusted to a value of 10 AU, and a 30-µl sample volume was applied to a Partisil-5 SAX RAC II column (Whatman) equilibrated with 5 mM KH_2_PO_4_. Bound metabolites were eluted with a binary gradient (1 ml/min, 36 column volumes) to 500 mM KH_2_PO_4_, and the absorbance monitored at 260 nm. The cell extracts obtained from cells expressing PezTΔC_242_ showed an additional peak eluting at the same retention time as purified UNAG-3P. Fractions containing this peak were pooled, desalted, and concentrated. Analysis by electrospray ionization tandem mass spectrometry demonstrated the presence of UNAG-3P in this sample (Table S1).

### MurA Activity/Inhibition Assay

The enolpyruvyl activity of *E. coli* MurA was monitored by coupling phosphate release to cleavage of fluorescent 7-methylguanosine (Sigma) by bacterial purine nucleoside phosphorylase (Sigma) [53]. Each reaction mixture of 400 µl contained 0.3 U purine nucleoside phosphorylase, 50 µM 7-methylguanosine, and varying concentrations of UNAG and UNAG-3P in buffer R2 (50 mM NaCl, 50 mM HEPES-NaOH [pH 7.5], 1 mM phosphoenolpyruvate). UNAG turnover was started by addition of MurA to 0.5 µM. The reactions were monitored by the decrease in fluorescence at 400 nm with excitation set to 300 nm in a FluoroMax-3 spectrofluorometer (Horiba Jobin Yvon) with excitation and emission bandwidths of 5 nm. In order to determine the *K*_i_ of UNAG-3P for MurA, initial velocities were plotted against the UNAG concentration and fitted globally assuming competitive inhibition of MurA by UNAG-3P using the equation
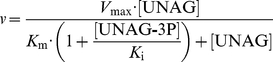
(1)where *V*_max_ is the maximal rate in fluorescence decrease at the given MurA concentration, *K*_m_ the Michaelis-Menten constant of UNAG for *E. coli* MurA, and *K*_i_ the inhibition constant of UNAG-3P. While the *K*_i_ for UNAG-3P converged to 7 µM, the best fit parameter for *K*_m_ was 15 µM, which is the same as reported previously [54].

## Supporting Information

Figure S1 **Response of** ***E. coli*** **cells grown as liquid culture to induction of PezTΔC_242_ expression.** (A) Phase contrast and fluorescence images of *E. coli* were recorded at *t* = 0 min, *t* = 30 min, and *t* = 60 min after induction after fluorescence staining as described in the Materials and Methods. (B) Representative close-ups of live and dead cells 30 min after induction. (C) Representative close-ups of cells surviving PezTΔC_242_ expression for 1 h. (D) Control cells 1 h after expression of nontoxic PezTΔC_242_ (D66T). (E) Growth curves and intracellular rRNA levels after expression of PezTΔC_242_ in *E. coli* BL21 (DE3) (upper panel) and RNase I–deficient *E. coli* D10 (DE3) (lower panel). In the RNase I–deficient *E. coli* D10 (DE3) strain, rRNA decay is absent. Note that all sample volumes applied to gel electrophoresis had equivalent A_260_ values. (F) *E. coli* BL21 (DE3) cells expressing nontoxic PezTΔC_242_ (D66T) show rRNA degradation only upon treatment with ampicillin (upper panel), but not after treatment with the translation inhibitor tetracycline (lower panel).
(TIF)

Figure S2 **The PezTΔC_242_ phosphoryltransferase activity is ATP dependent and specific for the N-acetylglucosamine moiety of UNAG.** (A) PezTΔC_242_ shows no turnover of UNAG in absence of ATP after 60 min incubation. (B) Control run of ATP and UNAG in absence of any enzyme. (C) PezTΔC_242_-dependent turnover of UNAG is observed only in the presence of ATP. (D) The nontoxic variant PezTΔC_242_ (D66T) shows no turnover of UNAG and ATP. Note that in this experiment a final protein concentration of 3 µM was used instead 1 µM as for all the other experiments. (E) PezTΔC_242_ shows no turnover of the nucleotide sugar UDP-glucose even after extended incubation for 12 h. (F) PezTΔC_242_ shows inefficient turnover of the UNAG stereoisomer UDP-*N*-acetylgalactosamine that can be detected after 12 h of incubation. The product of this reaction was not further characterized. Note that turnover of the same amount of UNAG by PezTΔC_242_ requires ∼30 min under the same conditions (data not shown).
(TIF)

Figure S3 **^1^H, ^13^C, and ^31^P NMR spectra of UNAG-3P.** ^1^H NMR spectra: δ_H_ 7.94 (d, *J*_HH_ = 8.1 Hz, 1H, H-U6), 5.97 (d, *J*_HH_ = 4.7 Hz, 1H, H-1′), 5.95 (d, *J*_HH_ = 8.1 Hz, 1H, H-U5), 5.53 (dd, *J*_HH_ = 3.3 Hz, *J*_HP_ = 7.3 Hz, 1H, H-1), 4.37–4.34 (m, 2H, H-2′, H-3′), 4.27–4.20 (m, 3H, H-4′, H-3, H-5′a), 4.18–4.15 (m, 1H, H-5′b), 3.99 (ddd, *J*_HH_ = 10.4 Hz, *J*_HH_ = 3.3 Hz, *J*_HP_ = 3.3 Hz, 1H, H-2), 3.94–3.91 (m, 1H, H-5), 3.84 (dd, *J*_HH_ = 12.5 Hz, *J*_HH_ = 2.3 Hz, 1H, H-6a), 3.78 (dd, *J*_HH_ = 12.5 Hz, *J*_HH_ = 4.4 Hz, 1H, H-6b), 3.67 (dd, *J*_HH_ = 10.1 Hz, *J*_HH_ = 8.7 Hz, 1H, H-4), 2.06 (s, 3H, H-8). ^13^C NMR spectra: δ_C_ 177.58 (C, C-7), 168.96 (C, C-U4), 154.57 (C, C-U2), 144.39 (C, C-U6), 105.39 (C, C-U5), 96.94 (CH, *J*_CP_ = 6.1 Hz, C-1), 90.96 (CH, C-1′), 85.98 (CH, *J*_CP_ = 9.2 Hz, C-4′), 76.85 (CH, *J*_CP_ = 5.3 Hz, C-3), 76.42 (CH, C-3′), 75.45 (CH, C-5), 72.65 (CH, *J*_CP_ = 0.8 Hz, C-4), 72.46 (CH, C-2′), 67.72 (CH_2_, *J*_CP_ = 5.6 Hz, C-5′), 63.00 (CH_2_, C-6), 55.61 (CH, *J*_CP_ = 8.7 Hz, *J*_CP_ = 5.8 Hz, C-2), 24.89 (CH_3_, C-8). ^31^P NMR spectra: δ_P_ 0.12 (P-3), −14.40 (d, *J*_PP_ = 20.3 Hz, P-5′), −15.93 (d, *J*_PP_ = 20.3 Hz, P-1).
(TIF)

Figure S4 **Overall architecture of the epsilon/zeta/UNAG complex.** (A) Ribbon representation of the heterotetrameric epsilon_2_zeta_2_ TA assembly in complex with UNAG. Helices of the epsilon antitoxin are colored in yellow, those of the zeta toxin in cyan. Strands within the zeta toxin are shown as magenta arrows. (B) The experimental electron density difference map before UNAG was modeled during refinement is shown as a mesh representation around the UNAG molecule contoured at 3σ.
(TIF)

Figure S5 **Presence of UNAG-3P impairs turnover of UNAG by MurA.** The MurA activity assay was performed as described in Materials and Methods. The reaction mix contained 20 µM UNAG and increasing concentrations of UNAG-3P as indicated.
(TIF)

Figure S6 **Growth in preconditioned LB medium leads to reduced cell size but has no major effect on PezTΔC_242_ expression levels.** (A) Phase contrast pictures of uninduced *E. coli* cells during exponential phase in fresh LB medium (upper panel) and nutritionally deprived medium (lower panel). Note that both cultures had an OD_600_ of 0.4. (B) PezTΔC_242_ shows similar expression levels in *E. coli* cultures grown in fresh LB medium or preconditioned LB. Protein expression was induced at an OD_600_ of 0.4. Samples with equivalent amounts of cells were analyzed by SDS-PAGE followed by Coomassie Blue staining (upper panel). (1) *E. coli* cells expressing PezTΔC_242_ in fresh medium, (2) *E. coli* cells expressing PezTΔC_242_ in preconditioned medium, and (3) *E. coli* cells expressing nontoxic PezTΔC_242_ (D66T) in fresh medium. Note that the band labeled with an asterisk, which is prominent exclusively in cells grown in preconditioned medium, was identified to be chloramphenicol acetyltransferase by peptide mass fingerprint analysis. The same samples were analyzed by a Western blot, which detected the C-terminal His_6_-tag of the PezT proteins (lower panel).
(TIF)

Table S1 **The** ***m/z*** **values for UNAG and UNAG-3P obtained by electrospray ionization.** Values were obtained with or without coupled tandem mass spectrometry for standard, substrate, and products formed by reaction by PezTΔC_242_ in vitro and in vivo. The observed monoisotopic *m/z* values (*m/z* obs.) and expected *m/z* values (*m/z* exp.) are from literature (http://www.massbank.jp/index.html, record PR100211) or calculated. Unless otherwise indicated, all ions are singly charged. In the identity column, +P indicates phosphorylation and −P indicates loss of phosphate from the phosphorylated product.
(DOC)

Table S2 **X-ray diffraction data and refinement statistics for crystals of the epsilon/zeta/UNAG complex.** *R*_free_ was calculated for 5% of the data. Numbers in parentheses represent values in the high-resolution shell.
(DOC)

Text S1 **Supplementary materials and methods.**
(DOC)

## Abbreviations

HPLC: high pressure liquid chromatograpy
LB: Luria broth
NMR: nuclear magnetic resonance
TA: toxin–antitoxin
UDP: uridine diphosphate
UNAG: uridine diphosphate-*N*-acetylglucosamine
UNAG-3P: uridine diphosphate-*N*-acetylglucosamine-3′-phosphate
