# Supplementary figures and images for: A Novel Mechanism of Programmed Cell Death in Bacteria by Toxin–Antitoxin Systems Corrupts Peptidoglycan Synthesis
Source: PLoS Biol. 2011 Mar 22;9(3):e1001033. doi: 10.1371/journal.pbio.1001033 (PMC3062530; doi:10.1371/journal.pbio.1001033)

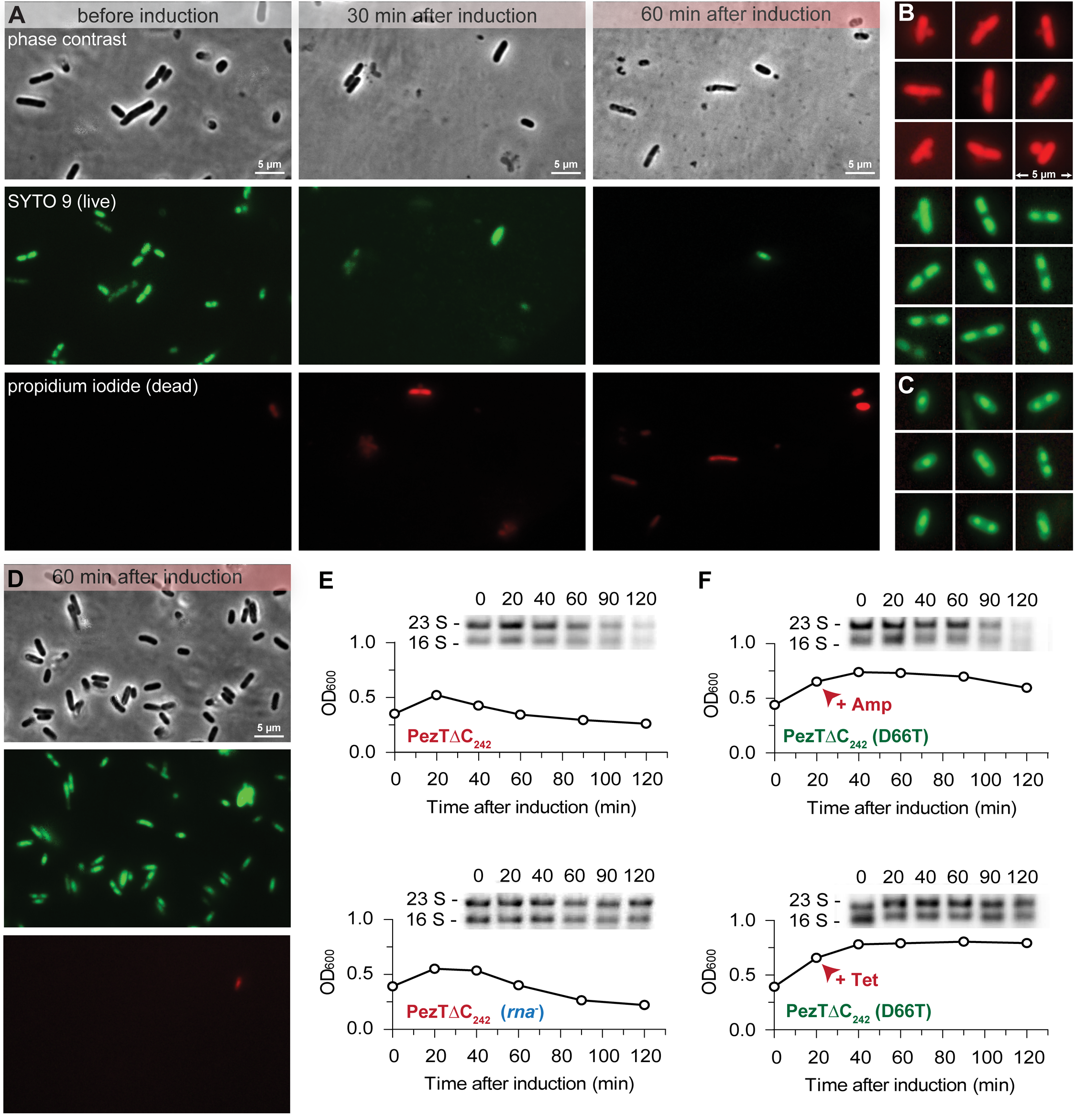

Supplement: Figure S1 — Response of E. coli cells grown as liquid culture to induction of PezTΔC242 expression. (A) Phase contrast and fluorescence images of E. coli were recorded at t = 0 min, t = 30 min, and t = 60 min after induction after fluorescence staining as described in the Materials and Methods. (B) Representative close-ups of live and dead cells 30 min after induction. (C) Representative close-ups of cells surviving PezTΔC242 expression for 1 h. (D) Control cells 1 h after expression of nontoxic PezTΔC242 (D66T). (E) Growth curves and intracellular rRNA levels after expression of PezTΔC242 in E. coli BL21 (DE3) (upper panel) and RNase I–deficient E. coli D10 (DE3) (lower panel). In the RNase I–deficient E. coli D10 (DE3) strain, rRNA decay is absent. Note that all sample volumes applied to gel electrophoresis had equivalent A260 values. (F) E. coli BL21 (DE3) cells expressing nontoxic PezTΔC242 (D66T) show rRNA degradation only upon treatment with ampicillin (upper panel), but not after treatment with the translation inhibitor tetracycline (lower panel). (TIF) [file pbio.1001033.s001.tif]

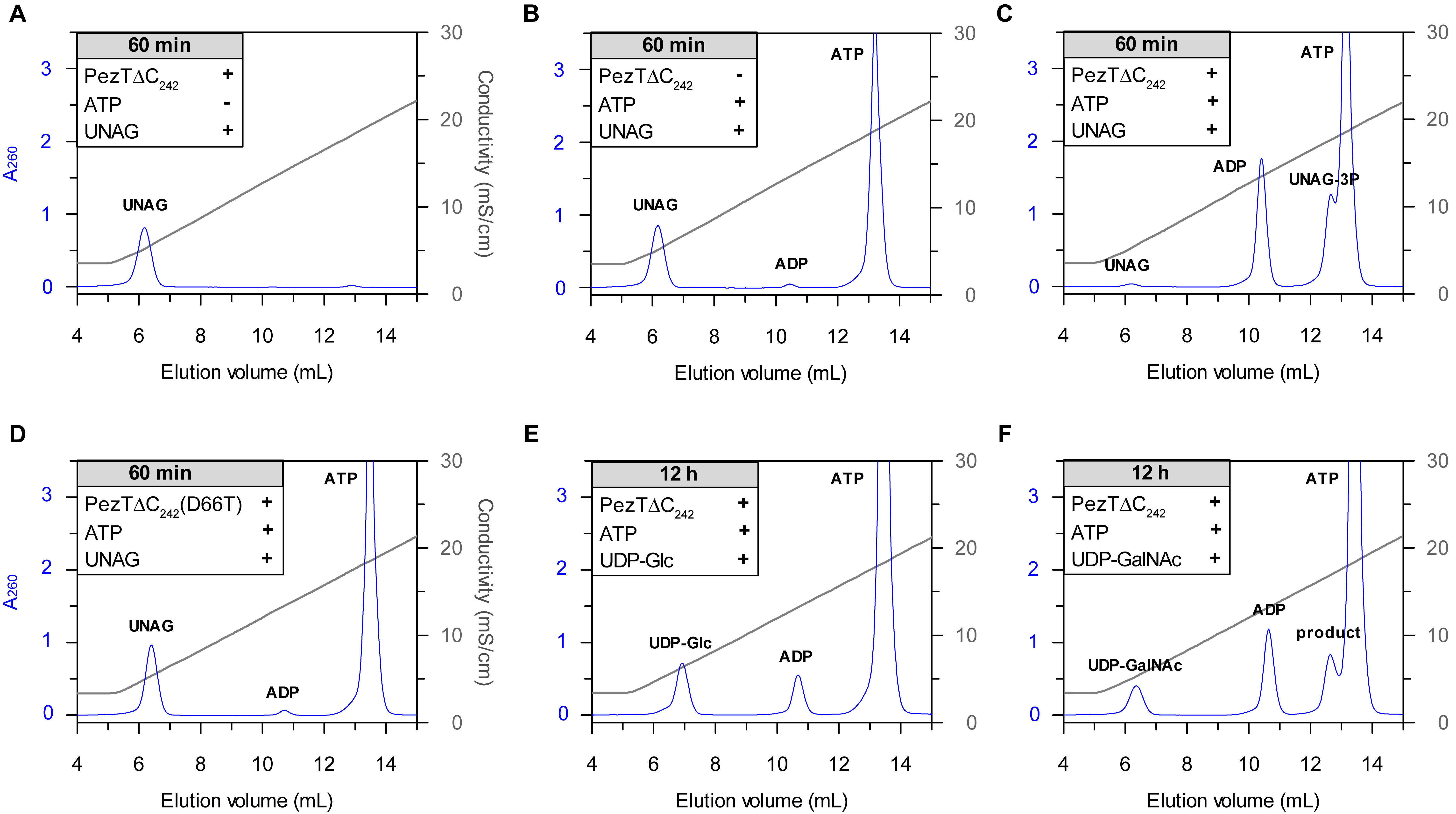

Supplement: Figure S2 — The PezTΔC242 phosphoryltransferase activity is ATP dependent and specific for the N-acetylglucosamine moiety of UNAG. (A) PezTΔC242 shows no turnover of UNAG in absence of ATP after 60 min incubation. (B) Control run of ATP and UNAG in absence of any enzyme. (C) PezTΔC242-dependent turnover of UNAG is observed only in the presence of ATP. (D) The nontoxic variant PezTΔC242 (D66T) shows no turnover of UNAG and ATP. Note that in this experiment a final protein concentration of 3 µM was used instead 1 µM as for all the other experiments. (E) PezTΔC242 shows no turnover of the nucleotide sugar UDP-glucose even after extended incubation for 12 h. (F) PezTΔC242 shows inefficient turnover of the UNAG stereoisomer UDP-N-acetylgalactosamine that can be detected after 12 h of incubation. The product of this reaction was not further characterized. Note that turnover of the same amount of UNAG by PezTΔC242 requires ∼30 min under the same conditions (data not shown). (TIF) [file pbio.1001033.s002.tif]

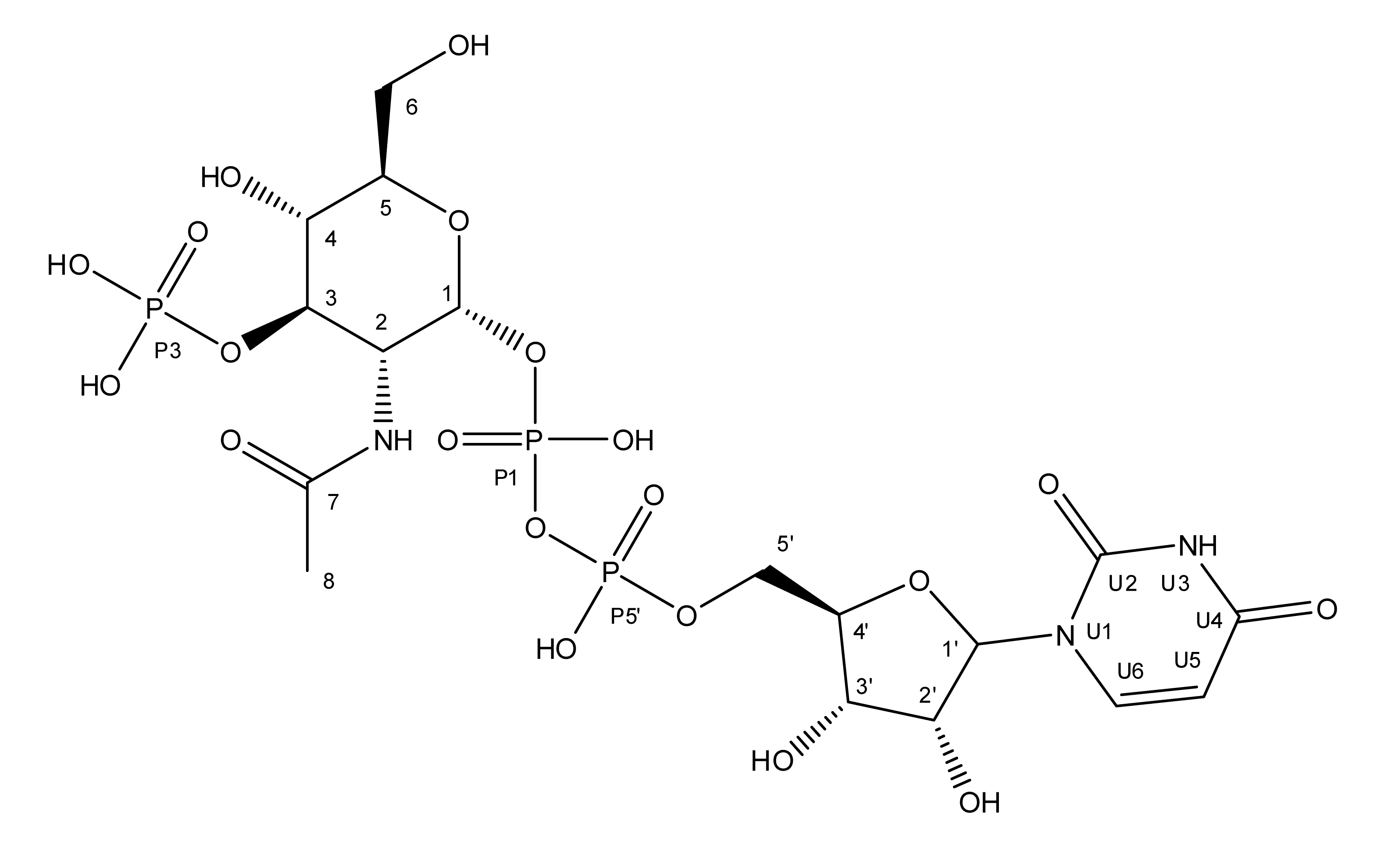

Supplement: Figure S3 — 1H, 13C, and 31P NMR spectra of UNAG-3P. 1H NMR spectra: δH 7.94 (d, J HH = 8.1 Hz, 1H, H-U6), 5.97 (d, J HH = 4.7 Hz, 1H, H-1′), 5.95 (d, J HH = 8.1 Hz, 1H, H-U5), 5.53 (dd, J HH = 3.3 Hz, J HP = 7.3 Hz, 1H, H-1), 4.37–4.34 (m, 2H, H-2′, H-3′), 4.27–4.20 (m, 3H, H-4′, H-3, H-5′a), 4.18–4.15 (m, 1H, H-5′b), 3.99 (ddd, J HH = 10.4 Hz, J HH = 3.3 Hz, J HP = 3.3 Hz, 1H, H-2), 3.94–3.91 (m, 1H, H-5), 3.84 (dd, J HH = 12.5 Hz, J HH = 2.3 Hz, 1H, H-6a), 3.78 (dd, J HH = 12.5 Hz, J HH = 4.4 Hz, 1H, H-6b), 3.67 (dd, J HH = 10.1 Hz, J HH = 8.7 Hz, 1H, H-4), 2.06 (s, 3H, H-8). 13C NMR spectra: δC 177.58 (C, C-7), 168.96 (C, C-U4), 154.57 (C, C-U2), 144.39 (C, C-U6), 105.39 (C, C-U5), 96.94 (CH, J CP = 6.1 Hz, C-1), 90.96 (CH, C-1′), 85.98 (CH, J CP = 9.2 Hz, C-4′), 76.85 (CH, J CP = 5.3 Hz, C-3), 76.42 (CH, C-3′), 75.45 (CH, C-5), 72.65 (CH, J CP = 0.8 Hz, C-4), 72.46 (CH, C-2′), 67.72 (CH2, J CP = 5.6 Hz, C-5′), 63.00 (CH2, C-6), 55.61 (CH, J CP = 8.7 Hz, J CP = 5.8 Hz, C-2), 24.89 (CH3, C-8). 31P NMR spectra: δP 0.12 (P-3), −14.40 (d, J PP = 20.3 Hz, P-5′), −15.93 (d, J PP = 20.3 Hz, P-1). (TIF) [file pbio.1001033.s003.tif]

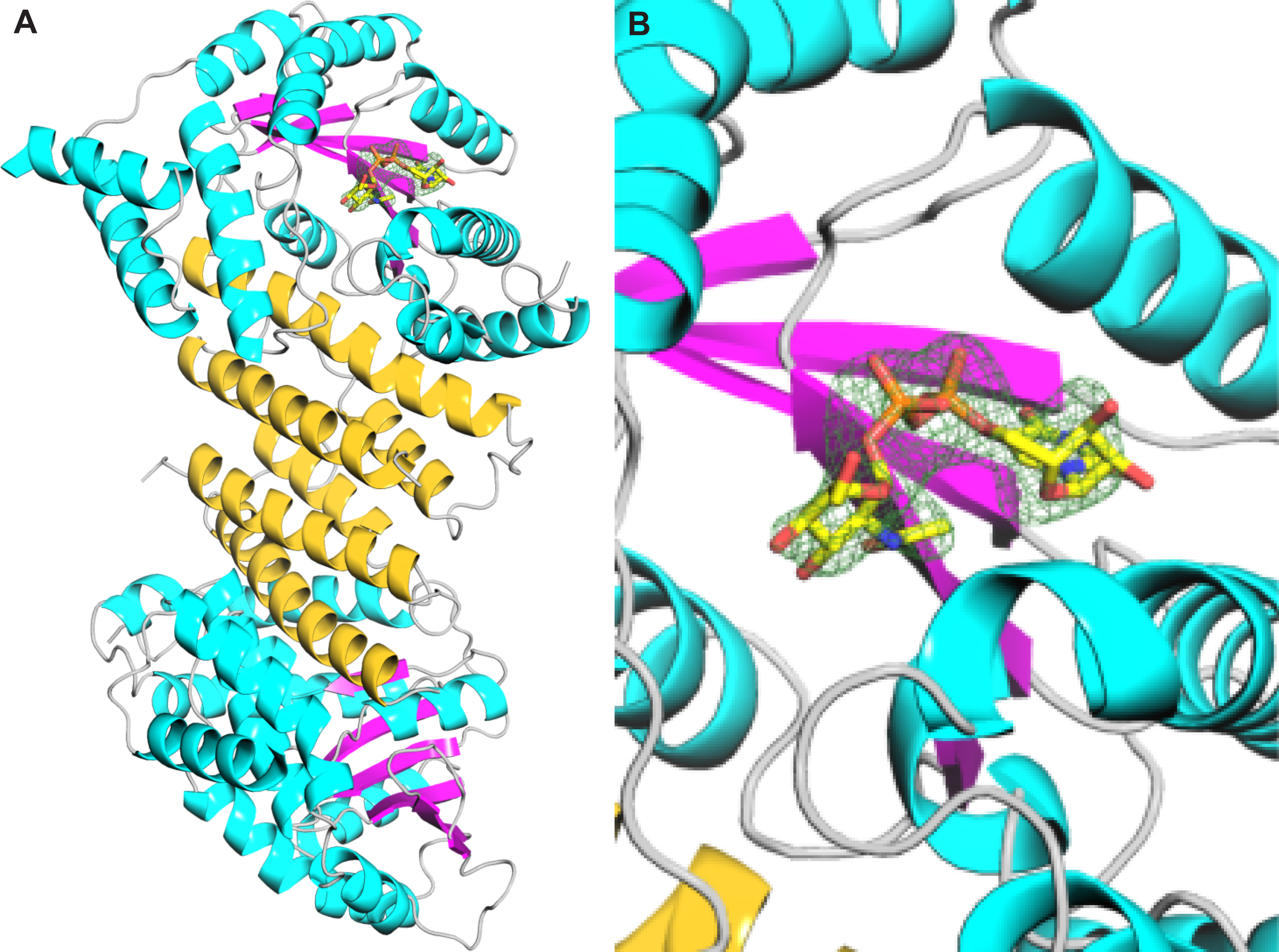

Supplement: Figure S4 — Overall architecture of the epsilon/zeta/UNAG complex. (A) Ribbon representation of the heterotetrameric epsilon2zeta2 TA assembly in complex with UNAG. Helices of the epsilon antitoxin are colored in yellow, those of the zeta toxin in cyan. Strands within the zeta toxin are shown as magenta arrows. (B) The experimental electron density difference map before UNAG was modeled during refinement is shown as a mesh representation around the UNAG molecule contoured at 3σ. (TIF) [file pbio.1001033.s004.tif]

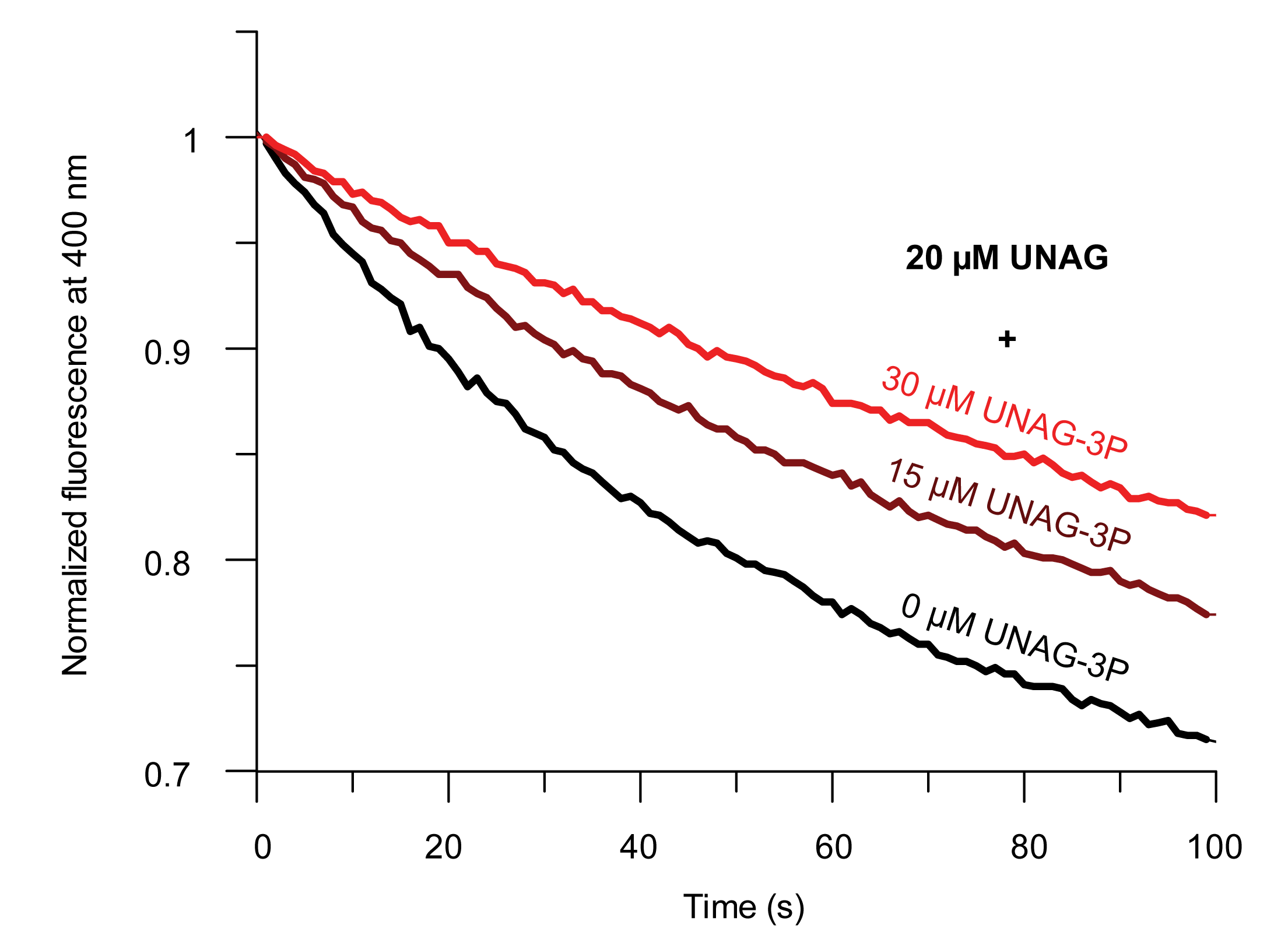

Supplement: Figure S5 — Presence of UNAG-3P impairs turnover of UNAG by MurA. The MurA activity assay was performed as described in Materials and Methods. The reaction mix contained 20 µM UNAG and increasing concentrations of UNAG-3P as indicated. (TIF) [file pbio.1001033.s005.tif]

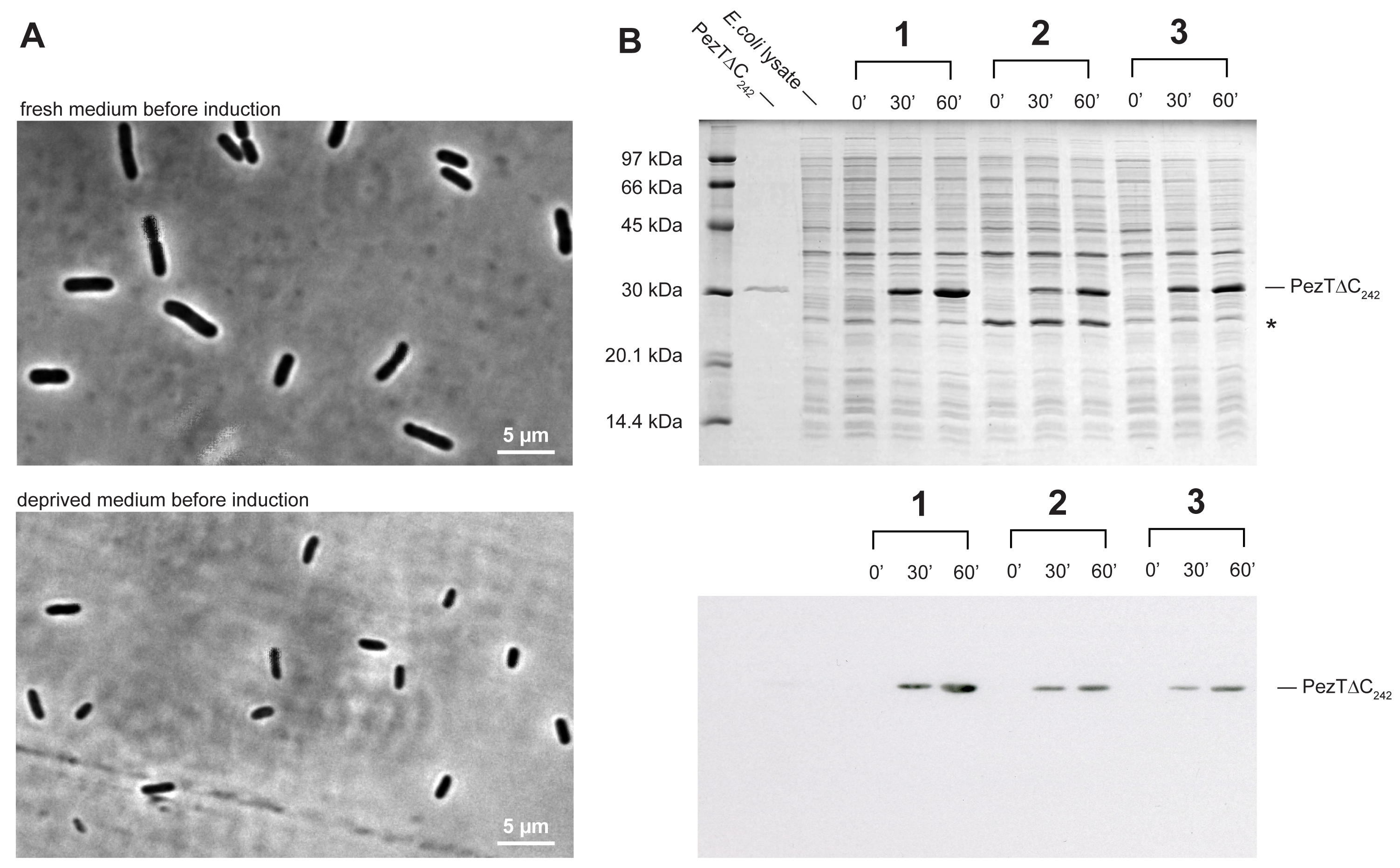

Supplement: Figure S6 — Growth in preconditioned LB medium leads to reduced cell size but has no major effect on PezTΔC242 expression levels. (A) Phase contrast pictures of uninduced E. coli cells during exponential phase in fresh LB medium (upper panel) and nutritionally deprived medium (lower panel). Note that both cultures had an OD600 of 0.4. (B) PezTΔC242 shows similar expression levels in E. coli cultures grown in fresh LB medium or preconditioned LB. Protein expression was induced at an OD600 of 0.4. Samples with equivalent amounts of cells were analyzed by SDS-PAGE followed by Coomassie Blue staining (upper panel). (1) E. coli cells expressing PezTΔC242 in fresh medium, (2) E. coli cells expressing PezTΔC242 in preconditioned medium, and (3) E. coli cells expressing nontoxic PezTΔC242 (D66T) in fresh medium. Note that the band labeled with an asterisk, which is prominent exclusively in cells grown in preconditioned medium, was identified to be chloramphenicol acetyltransferase by peptide mass fingerprint analysis. The same samples were analyzed by a Western blot, which detected the C-terminal His6-tag of the PezT proteins (lower panel). (TIF) [file pbio.1001033.s006.tif]
