# Supplementary material for: A Novel Mechanism of Programmed Cell Death in Bacteria by Toxin–Antitoxin Systems Corrupts Peptidoglycan Synthesis
Source: PLoS Biol. 2011 Mar 22;9(3):e1001033. doi: 10.1371/journal.pbio.1001033 (PMC3062530; doi:10.1371/journal.pbio.1001033)
Supplement: Table S1 — The m/z values for UNAG and UNAG-3P obtained by electrospray ionization. Values were obtained with or without coupled tandem mass spectrometry for standard, substrate, and products formed by reaction by PezTΔC242 in vitro and in vivo. The observed monoisotopic m/z values (m/z obs.) and expected m/z values (m/z exp.) are from literature (http://www.massbank.jp/index.html, record PR100211) or calculated. Unless otherwise indicated, all ions are singly charged. In the identity column, +P indicates phosphorylation and −P indicates loss of phosphate from the phosphorylated product. (DOC) [file pbio.1001033.s007.doc]

| **Sample** | **Mode** | ***m/z* obs.** | ***m/z* exp.** | **identity** |
| --- | --- | --- | --- | --- |
| UNAG | MS, + | 608.0882 | 608.0894 | intact molecule |
|  | MS/MS, + | 405.0091 | 405.01 | UDP fragment |
|  | MS/MS, + | 204.0866 | 204.0888 | sugar fragment |
|  |  |  |  |  |
| PezT *in vitro* product | MS, + | 688.0532 | 688.0557 | substrate +P |
| *m/z* 688 precursor | MS/MS, + | 284.052 | 284.0529 | sugar +P |
|  |  |  |  |  |
| PezT *in vitro* product (-2) | MS, - | 342.522 | 342.5161 | substrate +P |
| *m/z* 342.5 (-2) precursor | MS/MS, - | 402.9999 | 402.9944 | UDP |
| *m/z* 342.5 (-2) precursor | MS/MS, - | 282.0414 | 282.0372 | sugar +P |
| *m/z* 342.5 (-2) precursor | MS/MS, - | 606.0829 | 606.0738 | [substrate +P]-P |
|  |  |  |  |  |
| PezT *in vivo* product (-2) | MS, - | 342.5137 | 342.5161 | substrate +P |
| *m/z* 342.5 (-2) precursor | MS/MS, - | 402.9907 | 402.9944 | UDP |
| *m/z* 342.5 (-2) precursor | MS/MS, - | 282.0354 | 282.0372 | sugar +P |
| *m/z* 342.5 (-2) precursor | MS/MS, - | 606.0685 | 606.0738 | [substrate +P]-P |

[*http://www.massbank.jp/index.html. Record: PR100211](http://www.massbank.jp/index.html)
