# Supplementary material for: A Novel Mechanism of Programmed Cell Death in Bacteria by Toxin–Antitoxin Systems Corrupts Peptidoglycan Synthesis
Source: PLoS Biol. 2011 Mar 22;9(3):e1001033. doi: 10.1371/journal.pbio.1001033 (PMC3062530; doi:10.1371/journal.pbio.1001033)
Supplement: Table S2 — X-ray diffraction data and refinement statistics for crystals of the epsilon/zeta/UNAG complex. R free was calculated for 5% of the data. Numbers in parentheses represent values in the high-resolution shell. (DOC) [file pbio.1001033.s008.doc]

| ***Data collection*** |  |  |
| --- | --- | --- |
| Wavelength (Å) | 1 | |
| Space group | *P*2_1_2_1_2_1_ | |
| Unit cell parameter *a, b, c* (Å) | 59.8, 80.0, 195.2 | |
| Resolution range (Å) | 50.0 – 2.7 | (2.8 – 2.7) |
| Redundancy | 7.3 | -7.1 |
| Completeness (%) | 95.9 | -86.4 |
| *R_merge_* (%) | 4.8 | -54.8 |
| <I>/<sI> | 26.5 | -4.1 |
| Wilson B | 73.5 | |
|  |  |  |
| ***Refinement*** |  |  |
| Resolution range (Å) | 50.0 – 2.7 | |
| R_work_/R_free_ (%) | 21.0 / 26.3 | |
| Nr. of protein residues | 713 | |
| Nr. of bound ligands (UNAG, SO_4_^2-^) | 3 | |
| R.m.s.d. bond lengths (Å) | 0.009 | |
| R.m.s.d. bond angles (°) | 1.102 | |
| Average B factor (Å^2^) | 74.2 | |
| Ramachandran angles |  |  |
| Favored (%) | 98.01 | |
| Allowed (%) | 1.99 | |
| Disallowed (%) | 0 | |
