# Supplementary material for: A Novel Mechanism of Programmed Cell Death in Bacteria by Toxin–Antitoxin Systems Corrupts Peptidoglycan Synthesis
Source: PLoS Biol. 2011 Mar 22;9(3):e1001033. doi: 10.1371/journal.pbio.1001033 (PMC3062530; doi:10.1371/journal.pbio.1001033)
Supplement: Text S1 — Supplementary materials and methods. (DOC) [file pbio.1001033.s009.doc]

### Supplemental Material and Methods

###### Plasmid construction and protein purification

The plasmid containing the non-toxic PezTΔC_242_ (D66T) open-reading-frame with C-terminal His_6_-tag (pET28b(*pezTΔC_242_(D66T)*) was derived from the pET28b(*pezT(D66T)*) clone described previously [1] by PCR amplification with 5’-GCCGCA CTCGAGCAC-3’ as forward and 5’-AGATTTCTCGAGCGCTGCTGCCACCTGCA ACATCTCCTTC-3’as reverse primer using the QuikChange kit (Stratagene). The amplified vector DNA was linearized with XhoI and methylated template DNA was removed by a DpnI nuclease digest. The resulting vector DNA lacking a coding sequence for the last nine amino acids was recircularized using the Rapid DNA Ligation Kit (Fermentas), yielding pET28b(*pezTΔC_242_(D66T)*). Wild type coding sequence at the active site and thus toxicity of the construct was restored by site-directed mutagenesis of the codon triplet for Thr66 to Asp66 using the QuikChange kit with 5’-ATCATAGATGGTG ATAGTTTTCGTTCTC-3’ as forward and 5’-GAGAACGAAAACTATCACCATCTAT GAT-3’ as reverse primer, yielding pET28b(*pezTΔC_242_*). PezTΔC_242_ protein expression was induced by addition of IPTG to 1 mM at an OD_600_ = 1.0 for 1.5 h. Protein purification of PezT and antitoxin PezA was performed as described previously [1,2].

The *E. coli* MurA open reading frame was cloned from *E. coli* DH5α chromosomal DNA by PCR using the forward primer 5’-CACCATGGATAAATTTCGTGTTCAGGGGCC-3’ and the reverse primer 5’-TTATTCGCCTTTCACACGCTCAATATTTGC-3’. The PCR product was cloned into a pET151/D-TOPO vector (Invitrogen) following the supplier’s instructions, yielding the pET151(His_6_-*murA*) protein expression construct. MurA was purified using a HisTrap column (GE-Healthcare) equilibrated with buffer M1 (50 mM Tris-HCl pH 8.0, 200 mM NaCl, 50 mM NH_4_Cl, and 10 mM imidazole). After a high salt wash (50 mM Tris-HCl pH 8.0 and 1 M NaCl), the bound protein was eluted using buffer M1 containing additional 0.5 M imidazole. Pooled fractions containing the MurA protein were supplemented with TCEP and UNAG (final concentrations of 2 mM and 1 mM, respectively) to remove covalently bound phosphoenolpyruvate. After overnight dialysis against buffer M2 (50 mM Tris-HCl pH 8.0, 100 mM NaCl, 0.5 mM TCEP, and 1 mM EDTA), the protein was concentrated using an Amicon Ultra Centrifugal Filter Device (Millipore) and applied to a Superdex 75 column (GE-Healthcare) equilibrated with buffer M3 (50 mM HEPES-NaOH pH 7.5, 100 mM NaCl, 1 mM DTE, and 0.5 mM EDTA). Pooled fractions were concentrated and aliquots shock-frozen in liquid nitrogen and stored at -80°C. All protein concentrations were determined spectroscopically using calculated extinction coefficients at 280 nm.

###### Monitoring of intracellular rRNA levels

The RNase I deficient *E. coli* strain D10 (CGSC**#:** 6587) [3] was obtained from The Coli Genetic Stock Center (Yale). The λDE3 prophage was integrated into *E. coli* D10 using the λDE3 Lysogenization Kit (Merck) according to the supplier’s instructions. From the resulting *E. coli* D10 (DE3) strains, a single colony which showed strong induction after IPTG addition while lacking any apparent growth defect was chosen for all following experiments. Samples containing ribosomal RNA was prepared from either *E. coli* BL21-CodonPlus (DE3)-RIL or *E. coli* D10 (DE3) cells which had been transformed either with pET28(*pezTΔC_242_*) or pET28b(*pezTΔ_242_(D66T)*). Cells were grown in 100 mL LB medium and protein expression was induced at an OD_600_ = 0.4. Additional control cultures of E*. coli* BL21-CodonPlus (DE3)-RIL expressing non-toxic PezTΔC_242_ (D66T) were treated with either ampicillin or tetracycline 20 minutes after IPTG addition. Samples with a volume containing 0.3 OD_600_ of cells were withdrawn every 20 minutes and immediately shock-frozen and stored in liquid nitrogen. Subsequently, samples were thawed and subjected to a brief high-speed centrifugation step at 4°C. The cell pellet was resuspended in 30 µL proteinase K buffer (10 mM Tris-HCl pH 8.0, 3 mM CaCl_2_, and 1% (*w/v*) SDS) supplemented with 0.2 mg/mL proteinase K from *Tritirachium album*. Cellular proteins were removed by proteolytic digest for 20 minutes at 50°C. Total RNA was denatured by addition of 70 µL melting mix (5.3% (*v/v*) formaldehyde, 64.3% (*v/v*) formamide, 37.2 mM EDTA, 57 mM MOPS, 14.3 mM sodium acetate adjusted to pH 7.0 using NaOH), followed by two heat-denaturation steps of 5 min at 70°C and subsequently 2 min at 95°C. Samples were adjusted to an A_260_ of 5 AU and 10 µL of each sample was separated on 1% agarose gels containing 40 mM MOPS pH 7.0, 10 mM sodium acetate and 1 mM EDTA. The amount of intact 16S and 23S rRNA was assessed by conventional ethidium bromide staining.

###### Mass spectrometry

Samples were analyzed on a Bruker maXis hybrid quadrupole/ atmospheric pressure ionization orthogonal accelerated Time of Flight mass spectrometer, equipped with a reflectron and an Apollo II ESI source (Bruker Daltonics). Data were collected with the standard Bruker micrOTOF control version 3.0 SR1 Compass 1.3 SR2 software and analyzed with the Data Analysis version 4.0 SP3 ESI Compass 1.3 software. The instrument was calibrated with the appropriate components (*m/z* 118-1222) of the ESI-L standard (Agilent Technologies) and optimized for *m/z* range of ca. 100-1000. The average error (relative to published or calculated values) for all of the *m/z* values observed in these measurements was 11 ppm. Sample concentrations were adjusted to a final concentration of 10-60 µM in 50% acetonitrile, 49.9% water and 0.1% formic acid and were infused into the mass spectrometer at a flow rate of 3 µL/min using a syringe pump (KD Scientific). Samples were analyzed in both positive and negative polarity in MS and MS/MS modes (Table S1). MS/MS was performed with low energy CID, using various collision energies ranging from 5 to 50 V.

Peptide map fingerprinting was performed using standard procedures on peptides obtained by in-gel digestion of proteins with trypsin. The peptides were analyzed by MALDI-TOF using a Shimadzu Axima Performance mass spectrometer (Shimadzu/Kratos Analytical) in reflectron mode and identities of select peptides were confirmed by sequencing with high energy CID MS/MS on the same instrument. Protein identification was performed with the Mascot PMF or Mascot MS/MS search software (Matrix Science) using a hybrid taxonomy which includes all E. coli proteins and the specific proteins of interest.

###### Quantitative preparation of UNAG-3P

UNAG-3P used for MurA inhibition assays and NMR experiments was prepared by scaling up the analytical assay and using an excess of UNAG (1.25 mM) over ATP (1 mM). After incubation for 5 hours at 25°C, UNAG-3P was purified by anion exchange chromatography, desalted and concentrated in a Speed Vac Concentrator (Bachofer). For NMR experiments, H_2_O was replaced with D_2_O (99.96 % D, Euriso-Top) in two solvent exchange steps and freeze dried.

###### NMR Spectroscopy

Freeze-dried UNAG-3P (2 mg) was dissolved in 280 μl of D_2_O to give a NMR sample concentration of 10.5 mM. The NMR data were acquired at 298 K on a Bruker 600.13 MHz spectrometer equipped with a 5-mm ^13^C/^15^N/^31^P{^1^H} z-axis gradient cryogenic probe and a 500.13 MHz spectrometer with a 5-mm ^1^H{^13^C/BB} z-axis gradient probe. The NMR data were processed and analyzed with Bruker TopSpin 2.1 programs. ^1^H chemical shifts were referenced to the methyl resonance of DSS (3-(trimethylsilyl)-1-propanesulfonic acid sodium salt) which was determined applying the *substitution method* [4]. ^13^C and ^31^P chemical shifts were referenced indirectly to the ^1^H standard using published [5] conversion factors. Spectral resonance assignment was done with a combination of standard HSQC and HMBC experiments. *J*-coupling constants were obtained from 1D experiments after apodization of the FID with a Lorentz-to-Gauss transformation prior to Fourier transformation. Spectra assignments of Uridine-5’-diphosphate-*N*-acetyl-3’-phosphate-glucosamine are shown in Figure S3.

###### Structure Determination

Diffraction data were processed using XDS [6]. Phases were refined by molecular replacement methods with REFMAC [7] using the apo-structure of epsilon/zeta (PDB: 1GVN) as starting model. The model was improved by cycles of manual building using Coot [8] and refinement with REFMAC using TLS refinement [9]. Convergence was reached at an R-factor of 21.0 and the model quality was assessed using the MolProbity server [10]. Whereas one zeta molecule within the heterotetrameric assembly (chain D) contained a bound UNAG molecule, the binding site of the second toxin molecule was shielded in a crystal contact. The initial model already showed a strong electron density for the bound ligand (Figure S4) and thus one UNAG molecule was modeled unambiguously into the electron density map. Data and refinement statistics are given in Table S2. Illustrations were made using PyMOL (DeLano, W.L. The PyMOL Molecular Graphics System (2002) DeLano Scientific).

###### Growth modulation

Pre-conditioned LB medium was obtained by clearing an *E. coli* DH5α overnight culture, grown in absence of any antibiotics, by centrifugation and subsequent 0.2 μm sterile filtration. The pre-conditioned media was tested for any residual *E. coli* DH5α bacteria by incubation at 37°C for 3 h without inoculation.  For growth kinetics, *E. coli* BL21-CodonPlus (DE3)-RIL cells bearing pET28(*pezTΔC_242_*) were grown in fresh or pre-conditioned LB medium supplemented with kanamycin (50 µg/mL) at 37°C. Cell growth was measured by monitoring the OD_600_ at regular time intervals after induction with 1 mM IPTG and individual cultures were induced at different optical densities. Levels of protein expression of different PezTΔC_242_ variants in different media were estimated by resuspending pellets containing 0.2 OD_600_ of cells in 80 µL SDS loading buffer (10 mM Tris-HCl pH 7.0, 2% (*w/v*) SDS, 0.02 % (*w/v*) bromphenol blue, 0.2 % (*v/v*) β-mercaptoethanol, 2.8 % (*w/v*) DTE, 2% (*v/v*) glycerol) and subsequently analyzed by SDS-PAGE and conventional Coomassie Blue staining. The identity of the protein bands was verified by western blot analysis using the Penta-His HRP Conjugate Kit (Qiagen) and chemoluminescence methods according to the supplier’s protocol. In other experiments, the proteins were identified by peptide map fingerprinting and MALDI-TOF mass spectrometry (results not shown).

### Supplemental References

1. Khoo SK, Loll B, Chan WT, Shoeman RL, Ngoo L, et al. (2007) Molecular and structural characterization of the PezAT chromosomal toxin-antitoxin system of the human pathogen Streptococcus pneumoniae. J Biol Chem 282: 19606-19618.

2. Mutschler H, Reinstein J, Meinhart A (2010) Assembly dynamics and stability of the pneumococcal epsilon zeta antitoxin toxin (PezAT) system from *Streptococcus pneumoniae*. J Biol Chem 285: 21797-21806.

3. Gesteland RF (1966) Isolation and characterization of ribonuclease I mutants of *Escherichia coli*. J Mol Biol 16: 67-84.

4. Harris RK, Becker ED, Cabral de Menezes SM, Goodfellow R, Granger P (2001) NMR nomenclature. Nuclear spin properties and conventions for chemical shifts (IUPAC Recommendations 2001). Pure Appl Chem 73: 1795-1818.

5. Markley JL, Bax A, Arata Y, Hilbers CW, Kaptein R, et al. (1998) Recommendations for the presentation of NMR structures of proteins and nucleic acids (Recommendations 1998). Pure Appl Chem 70: 117-142.

6. Kabsch W (1993) Automatic processing of rotation diffraction data from crystals of initially unknown symmetry and cell constants. Journal of Applied Crystallography 26: 795-800.

7. Murshudov GN, Vagin AA, Dodson EJ (1997) Refinement of macromolecular structures by the maximum-likelihood method. Acta Crystallogr D Biol Crystallogr 53: 240-255.

8. Emsley P, Lohkamp B, Scott WG, Cowtan K (2010) Features and development of Coot. Acta Crystallogr D Biol Crystallogr 66: 486-501.

9. Winn MD, Isupov MN, Murshudov GN (2001) Use of TLS parameters to model anisotropic displacements in macromolecular refinement. Acta Crystallogr D Biol Crystallogr 57: 122-133.

10. Chen VB, Arendall WB, 3rd, Headd JJ, Keedy DA, Immormino RM, et al. (2010) MolProbity: all-atom structure validation for macromolecular crystallography. Acta Crystallogr D Biol Crystallogr 66: 12-21.
